# Self-Acupressure for Fatigue in Patients Surviving Ovarian Cancer: A Randomized Clinical Trial

**DOI:** 10.1001/jamanetworkopen.2025.56357

**Published:** 2026-02-05

**Authors:** Suzanna M. Zick, Dongru Chen, Richard E. Harris, Grant Kruger, Amy Runyon, Ananda Sen, Sara Snyder, Celeste Leigh Pearce

**Affiliations:** 1 Department of Family Medicine and Nutritional Sciences, University of Michigan, Ann Arbor; 2 Department of Family Medicine, University of Michigan Medicine, Ann Arbor; 3 Department of Anesthesiology, University of Michigan, Ann Arbor; 4 Now with Susan Samueli Integrative Health Institute, University of California Irvine, School of Medicine; 5 Department of Mechanical Engineering and Anesthesiology, University of Michigan Medicine, Ann Arbor; 6 Department of Family Medicine and Biostatistics, University of Michigan Medicine, Ann Arbor; 7 Department of Epidemiology, University of Michigan, Ann Arbor

## Abstract

**Importance:**

Fatigue is a burdensome effect of ovarian cancer that is associated with poor sleep and quality of life. Self-acupressure is recommended in clinical guidelines but has substantial barriers to implementation. Use of a mobile app may address these barriers.

**Objective:**

To investigate whether 6 weeks of true self-acupressure (TSA), learned via a mobile app, improves cancer fatigue, sleep, and quality of life in women with ovarian cancer compared with sham self-acupressure (SSA) and usual care (UC) and whether changes are sustained during an 18-week washout period.

**Design, Setting, and Participants:**

This phase 3 single-blind randomized clinical trial was conducted from October 2019 to December 2023. Data collection ended in November 2024. Participants included ovarian cancer survivors who were fatigued (based on a Brief Fatigue Inventory [BFI] score ≥4) and who were recruited from tumor registries and social media.

**Intervention:**

Randomization (1:1:1) to 6 weeks of TSA or SSA, taught via mobile app, or UC.

**Main Outcomes and Measures:**

The primary outcome was the change in the BFI from baseline to week 6. Secondary analyses were the BFI score at week 24 and sleep disturbance (based on the Pittsburgh Sleep Quality Index) and quality of life (based on the Functional Assessment of Cancer Therapy-Ovarian) administered at baseline and at weeks 6, 12, and 24.

**Results:**

Among the 360 participants who were screened, 171 women were randomized (mean [SD] age, 56 [12] years). Of the 160 participants who were allocated to the arms, 53 (33.1%) received TSA, 56 (35.0%) received SSA, and 51 (31.9%) received UC. Of these, the proportion achieving a clinically normal fatigue level at the end of treatment was 58.5% for the TSA arm, 51.1% for the SSA arm, and 17.6% for the UC arm. At 6 weeks, the BFI change scores were significantly better in the TSA arm but not in the SSA arm when they were compared with the UC-only arm (TSA vs UC: adjusted mean difference, −1.23 [95% CI, −2.17 to −0.29] and SSA vs UC: adjusted mean difference, −0.91 [95% CI, −1.83 to 0.02]). TSA and SSA change scores did not differ significantly from one another. The relative benefit of self-acupressure compared with UC on fatigue persisted at 24 weeks (TSA vs UC: mean difference, −1.38 [95% CI, −2.36 to −0.41] and SSA vs UC: mean difference, −0.97 [95% CI, −1.93 to −0.02]). Neither TSA nor SSA was significantly different than UC or each other for sleep quality. Only TSA significantly improved quality of life vs UC (odds ratio, 2.85 [95% CI, 1.20 to 6.80]). Neither true nor sham self-acupressure led to any adverse events.

**Conclusions and Relevance:**

In this randomized clinical trial, TSA and SSA significantly reduced fatigue compared with UC, and these changes were both clinically meaningful and sustained. No impact was observed on sleep quality. Self-acupressure, taught via a mobile app, offered a safe and low-cost option for managing cancer fatigue.

**Trial registration:**

ClinicalTrials.gov Identifier: NCT03763838

## Key Points

**Question:**

Does true self-acupressure, learned through a mobile app, improve cancer-related fatigue in women with ovarian cancer compared with sham self-acupressure and usual care?

**Findings:**

In this randomized clinical trial of 171 women, the proportion achieving a clinically normal fatigue level at the end of treatment was 58% for true self-acupressure, 51% for sham self-acupressure, and 18% for usual care, representing a significant difference between true or sham self-acupressure vs usual care.

**Meaning:**

The findings suggest that self-acupressure, learned through a mobile app, offers a possible low-cost option for managing cancer-related fatigue in women diagnosed with ovarian cancer.

## Teaser

This randomized clinical trial investigates whether true self-acupressure, learned through a mobile app, improves cancer-related fatigue in women surviving ovarian cancer compared with sham self-acupressure and usual care.

## Introduction

Approximately 250 000 women are living with ovarian cancer in the US.^1^ Cancer-related fatigue (CRF) is a commonly reported symptom in these women,^2,3^ with a recent meta-analysis finding that the mean prevalence of acute CRF was 77%, and chronic CRF ranged from 22% to 32.7%.^4^ In ovarian cancer, CRF has been associated with higher levels of chronic pain,^5,6^ a lower quality of life,^7,8^ and reduced participation in self-care activities.^9,10^ While the etiology of CRF is unknown, recent research in cancer survivors suggests that CRF may involve pathology within the brain, and thus treatments such as acupressure that target the brain may have promise for treating fatigue.^11,12^

Our team’s prior research has shown that self-acupressure is an effective and safe method to manage CRF in female cancer survivors,^13,14^ and the most recent American Society of Clinical Oncology-Society for Integrative Oncology clinical guidelines for CRF recommends self-acupressure for posttreatment CRF.^15^ However, to our knowledge, acupressure studies for CRF have been almost exclusively in breast cancer survivors, which may not be applicable to ovarian cancer survivors, in whom the etiology and risk factors for CRF may be different.^16^ Also, previous research used acupressure educators to teach women acupressure^13,14^; these approaches create substantial barriers to accessibility and scalability. A mobile app that teaches self-acupressure could help to address the barriers. To address these gaps we conducted a randomized clinical trial comparing true self-acupressure (TSA) with sham self-acupressure (SSA), both taught via a mobile app, and usual care (UC) to determine if this is an intervention that decreases CRF in ovarian cancer survivors.

## Methods

### Trial Design and Oversight

We conducted a remotely administered 3-arm randomized clinical trial design to compare the effects of 6 weeks of once-daily TSA (intervention) with SSA (control arm 1), both learned through a mobile app plus acupressure device, and with UC (control arm 2) on CRF in ovarian cancer survivors. The trial protocol is available in Supplement 1. The trial was conducted between October 2019 and December 2023; data collection ended in November 2024. The trial included 5 remote study visits: screening, baseline, week 6 (end of intervention), week 12, and week 24 (end of washout phase). The study was approved by the institutional review boards of the University of Michigan Medical School, the US Department of Defense, the Michigan Department of Health and Human Services, the California Cancer Registry, and the North Carolina State Center for Health Statistics. Electronic written informed consent was obtained from all participants prior to their enrollment. The study design has been previously described^17^ and followed the Consolidated Standards of Reporting Trials (CONSORT) reporting guideline.

### Participants

Participants were identified through the Michigan Department of Health and Human Services, Michigan Cancer Surveillance Program; the University of Michigan Medicine’s Rogel Cancer Center’s Cancer Registry; the Los Angeles Cancer Surveillance Program; the North Carolina Central Cancer Registry; and several ovarian cancer survivor groups via social media. Eligible participants were women aged 21 years or older with a diagnosis of ovarian cancer at any stage or histotype, who had initiated cancer treatment such as surgery or chemotherapy at least 4 weeks prior to study enrollment. Participants had to report their current CRF (based on the Brief Fatigue Inventory [BFI] ≥4)^18^ that started at or after their cancer diagnosis and to have had no other planned intervention for CRF except stable medication or therapies. Exclusion criteria included untreated mood disorders, anemia, or hypothyroidism; acupuncture or acupressure within the past year; and current pregnancy. Race and ethnicity categories included Asian, Black or African American, Hispanic, Native Hawaiian or Other Pacific Islander, non-Hispanic White, 2 or more races, and unknown. Race and ethnicity were self-reported by participants and were included in the study to evaluate whether there was a representative sample of ovarian cancer survivors.

### Intervention

**Figure 1.**
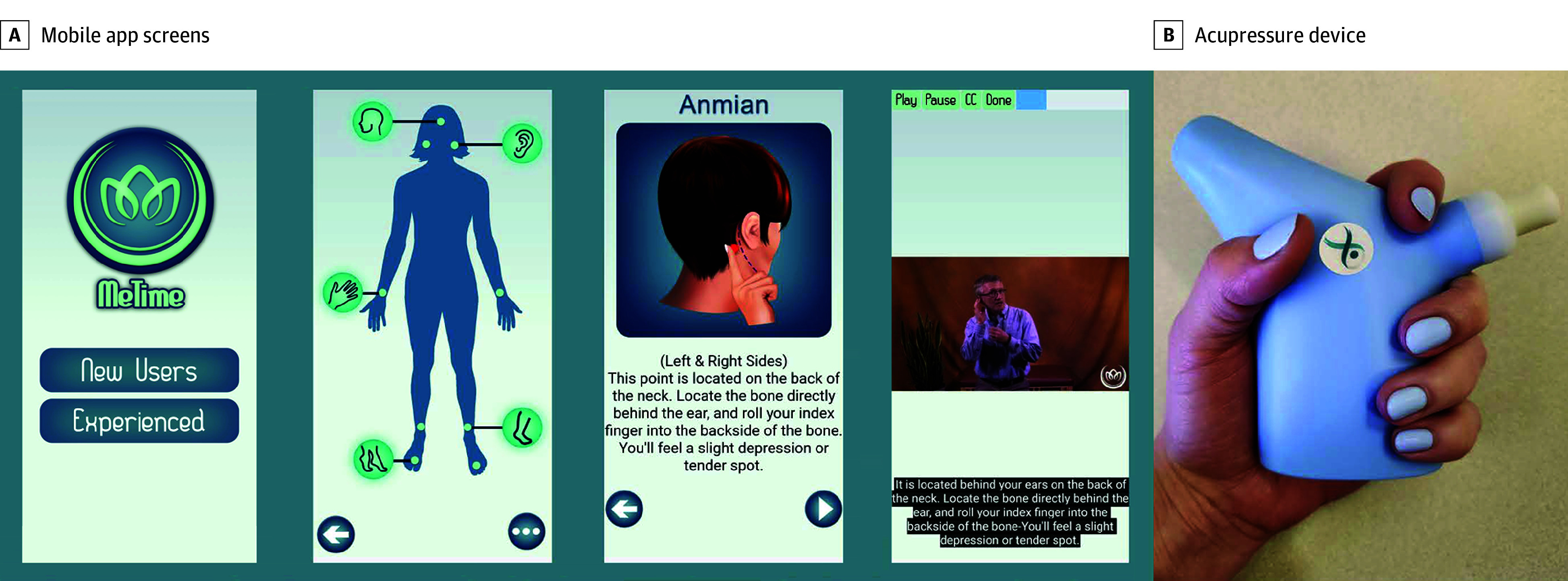
Mobile App Screen Shots and Acupressure Device A, Screen shots from the mobile app (MeTime) showing the welcome image (first screen), point map (second screen), and an illustration (third screen) and video (fourth screen) of how to find the anmian acupoint. B, The acupressure device being held in one of its possible positions. Participants were told to place the tip of the device on each acupoint and use either clockwise or counterclockwise circular motions to stimulate the acupoint. The device delivered vibrational cues when a participant exceeded 3 kg/mm^2^ to minimize excessive force and bruising. The device was designed by Arbor Medical Innovations, in association with cancer survivors, to be comfortable to use and to minimize any strain in the hand or fingers from applying the acupressure.

This was a remotely delivered intervention. The TSA intervention was learned via the acupressure app (MeTime), developed by S.M.Z. and R.E.H., and the acupressure device, developed by S.M.Z., R.E.H., and G.K.,^19^ in association with cancer survivors with CRF^17^ (Figure 1). The acupressure app was installed on computer tablets, and both the tablet and acupressure device were mailed to participants along with instructions on how to use the device, how to turn on and charge the tablet, and how to locate and open the app. No other information was provided on how to perform acupressure or how to use the app. Instead, participants were instructed by illustrations and videos within the app on acupressure point (acupoint) location, amount of pressure to apply, how to correctly stimulate acupoints, and the amount of time and number of weeks to conduct acupressure. Participants randomized to the TSA arm were told to perform daily self-acupressure on 5 specific acupoints (yin tang, anmian, heart 7, spleen 6, and liver 3) for 3 minutes per point for 27 minutes daily for 6 weeks. The SSA arm used the same app and device but applied pressure to 5 nontherapeutic points located in the same areas of the body and for the same length of time as the true acupoints (eFigure in Supplement 2). Both arms were told to stop acupressure after week 6. The UC arm received no interventions and was instructed to continue care for their CRF as directed by their health care team. Acupressure arms also received UC for CRF.

### Randomization and Blinding

Randomization was performed by the study biostatistician (A.S.) using a computer-generated algorithm in blocks of 6. The study’s information technology support person placed computer tablets—preloaded with either the true acupressure app or the sham acupressure app and the acupressure devices—into shipping boxes according to our randomization code. The outside of each shipping box was labeled with color-coded identifiers. This ensured that only our information technology support person, who was not responsible for collecting or analyzing study data, was unblinded. All other study staff and researchers (including S.S.) were blinded until after completion of data analyses (assisted by D.C. and A.R.). Participants in the UC arm were not blinded; however, the self-acupressure arms were blinded as to which acupressure treatment arm they were randomized. Eligible participants were randomized into the next participant study identifier in sequential order.

### Outcome Measures

The primary outcome CRF was measured using the BFI at week 6.^18^ The BFI is a validated instrument with a Cronbach α > 0.95 in patients with cancer that correlates well with other fatigue measures.^20^ It assesses the severity and impact of CRF on daily functioning over the past 24 hours. The BFI consists of 9 items, each scored on a 0-to-10 scale, with the final score calculated as the mean of completed items.^18^ Scores 4 or more indicate clinically relevant CRF, 6 or more indicate severe CRF, and a 1.33-point reduction or a drop below 4 is considered clinically meaningful.^21^

Secondary outcomes were to evaluate the proportion of participants who no longer had clinically relevant CRF (BFI <4 at weeks 6, 12, and 24) and the persistence of self-acupressure’s effect on CRF, sleep quality, and quality of life. The persistence of self-acupressure’s effect was evaluated with the BFI at weeks 12 and 24. Sleep quality and disturbance were assessed using the Pittsburgh Sleep Quality Index (PSQI), a 19-item validated tool that measures sleep disturbances over the past month including sleep quality, latency, duration, habitual sleep efficiency, disturbances, use of sleep medication, and daytime dysfunction. The PSQI global score is a sum of the 7 component scores and ranges from 0 to 21, with higher scores indicating worse sleep quality.^22^ A PSQI score of more than 6 is suggestive of poor sleep quality,^23^ and a 3-point reduction represents a minimal clinically important difference with scores of 5 or less indicating remission of sleep disturbance.^24,25,26^ Quality of life was measured using the Functional Assessment of Cancer Therapy-Ovarian (FACT-O) Trial Outcome Index, which is a multidimensional 26-item summary score instrument that includes the 27-item FACT-General core and 12 ovarian cancer-specific items. Higher scores indicate a better quality of life,^27^ and a clinical meaningful difference is considered to be a change of 5 to 7 points.^28^ Both the PSQI and FACT-O were administered at weeks 0, 6, 12, and 24.

### Adverse Effects, Adherence, and Fidelity

Adverse events were graded according to the National Cancer Institute Common Toxicity Criteria, version 4.03.^29^ Adherence to acupressure was assessed in daily study logbooks, in which participants were asked to record how many minutes they performed acupressure. Participant fidelity was assessed at week 6 via a video call, in which participants were asked to identify the location of their acupoints and to demonstrate the acupressure stimulation technique and the amount of pressure used on their acupoints. The number of correct responses (0 to 12) was recorded as yes or no and converted to the percentage of correct answers from 0% to 100%.^30^

### Sample Size and Power

Our primary hypothesis was that TSA would improve CRF in ovarian cancer survivors compared with SSA or UC at week 6. The power to detect differences between the acupressure arms and the UC arm was determined using simulation by a mixed-effects model with a group, visit, group-by-visit interaction, and a random subject effect between baseline and week 6. Based on our prior research, the mean BFI at baseline was assumed to be 5.5, whereas values at the end of treatment were assumed to be 4.5 for the UC arm, 3.5 for the SSA arm, and 2.0 for the TSA arm.^13,14^ The between-subject variance was assumed to be 4 at all time points, whereas the variance of the random subject component was taken to be 4 also (yielding an intraclass correlation of 0.5).^13,14^ For this configuration, the power for detecting a significant visit-by-group interaction was greater than 95% with a sample size of 50 evaluable participants per treatment arm (150 total) and a 5% level of significance. Such a model can detect a post hoc difference between the TSA and each of the other 2 arms with more than 90% power at a reduced 2-sided α = .025 level of significance.

### Statistical Analysis

An intention-to-treat analysis was used to report all available data. Differences between arms on baseline characteristics, adverse events, fidelity, and adherence were tested using analysis of variance or a Pearson χ^2^ test, as appropriate.

The primary outcome of CRF between TSA and SSA and between TSA and UC at week 6 was examined using linear mixed-effects models with group, week (categorical), and group-by-week interaction; a random effect for participants; and an unstructured covariance matrix. The test for overall significance of fixed effects for the linear mixed-effects model was the *F* test. Pairwise comparisons were conducted using estimated marginal means with a post hoc adjustment (Bonferroni). The same analytical method with the linear mixed-effects model was used to investigate secondary outcomes of CRF at 12 and 24 weeks and the PSQI and FACT-O at 6, 12, and 24 weeks.

To examine the proportion of participants who no longer had clinically relevant CRF (BFI <4 at weeks 6, 12, and 24), whose sleep quality was considered normal (PSQI ≤5), or whose quality of life had meaningfully improved (a ≥5-point increase on the FACT-O Trial Outcome Index), we conducted a logistic regression model for each outcome reporting the odds ratios (ORs) and 95% CIs.

Multiple testing for CRF analyses (changes in BFI scores and BFI clinical responders) was addressed using a Bonferroni adjustment, with a 2-sided *P* ≤ .025 considered significant. For all other analyses, a 2-sided *P* ≤ .05 was considered statistically significant. No adjustment was made for exploratory outcomes, which were considered hypothesis generating. All statistical tests were performed using SPSS Statistics, version 30.0 (IBM Inc).

## Results

**Figure 2.**
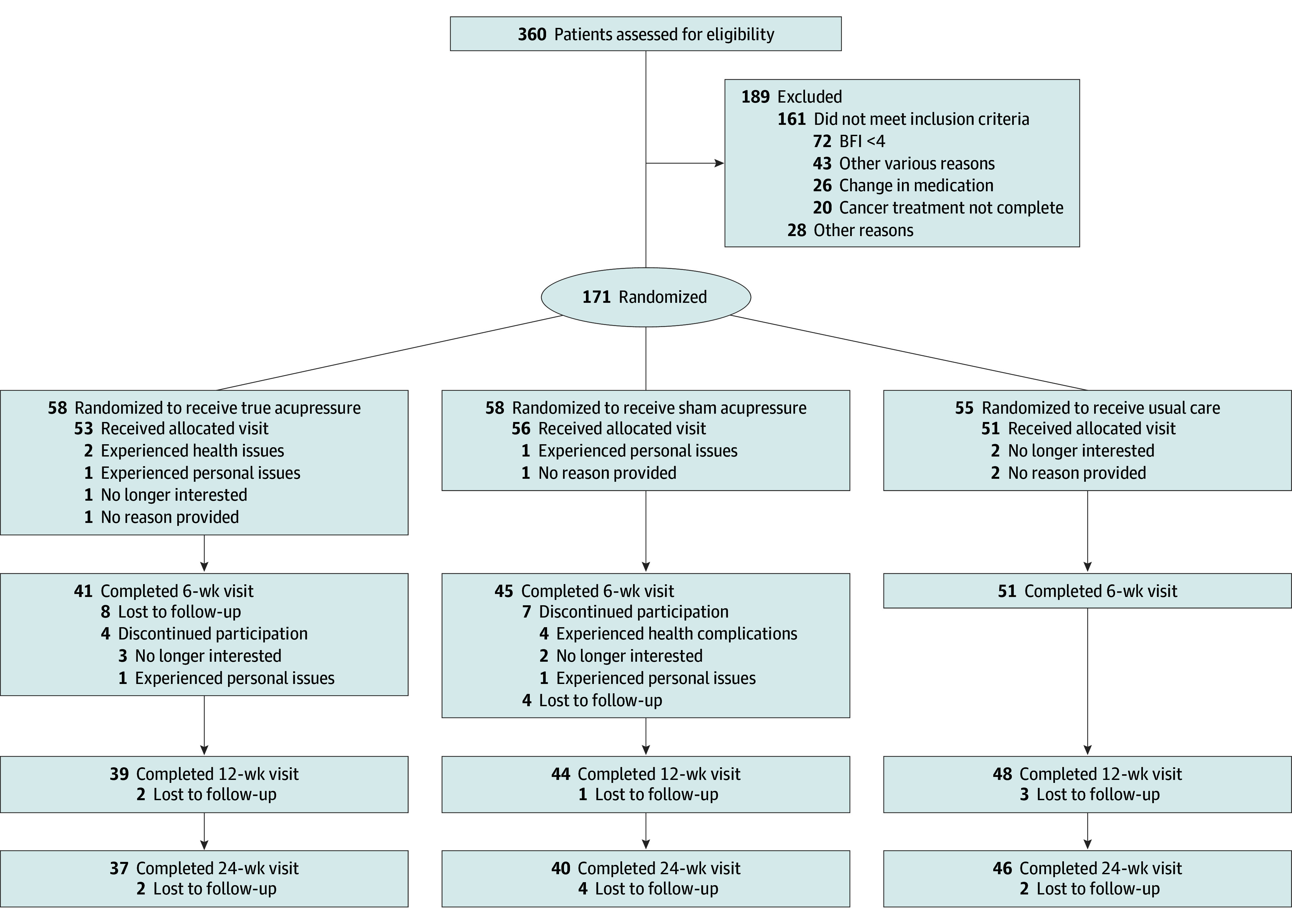
Patient Flow Diagram BFI indicates Brief Fatigue Inventory.

We screened 360 women, of whom 189 were not eligible; the primary reason for ineligibility was not being fatigued (BFI <4; 72 of 161 participants [44.7%]). We randomized 171 women (mean [SD] age, 56 [12] years): 58 (33.9%) to the TSA arm, 58 (33.9%) to the SSA arm, and 55 (32.2%) to the UC arm. Of the 160 participants who were allocated to the arms, 53 (33.1%) received TSA, 56 (35.0%) received SSA, and 51 (31.9%) received UC. A total of 137 women completed the week-6 visit: 41 (29.9%) in the TSA arm, 45 (32.8%) in the SSA arm, and 51 (37.2%) in the UC arm. At week 24, 123 participants remained: 37 (30.1%) in the TSA arm, 40 (32.5%) in the SSA arm, and 46 (37.4%) in the UC arm. More women withdrew from the self-acupressure arms—29% in the TSA arm and 22% in the SSA arm—compared with 7% in the UC arm. Reasons for withdrawals and the number of participants completing the primary and secondary end points at each visit (baseline and weeks 6, 12, and 24) are presented in Figure 2.

Of the 171 total participants, 4 (2%) were Asian, 4 (2%) were Black or African American, 10 (6%) were Hispanic, 2 (1%) were Native Hawaiian or Other Pacific Islander, 147 (86%) were non-Hispanic White, 1 (<1%) was of 2 or more races, and 3 (2%) were of unknown race or ethnicity; and 81 (47%) had stage III or IV disease at diagnosis. High-grade serous cancer was the most common histotype (45 [36%]). The mean (SD) of women who had been diagnosed over 4 years prior to study enrollment was 48 (12) months. Sociodemographic and clinical characteristics by study arm are presented in the eTable in Supplement 2.

### Impact on Cancer Fatigue

Women were moderately fatigued upon entry into the study (mean [SD] BFI, 5.51 [1.66]). At week 6, there was a statistically significant reduction in CRF between TSA and UC (mean difference, −1.23 [95% CI, −2.17 to −0.29]; *P* = .005) in favor of the TSA arm, but there was no statistically significant difference between the TSA and the SSA arms (mean difference, −0.32 [95% CI, −1.28 to 0.64]; *P* = .99). The BFI change scores were significantly better in the TSA arm but not in the SSA arm (adjusted mean difference, −0.91 [95% CI, −1.83 to 0.02]; *P* = .06) when they were compared with the UC-only arm. The relative benefit of TSA compared with UC on fatigue persisted at 24 weeks.

The week-24 visit had similar findings, with a statistically significant reduction in CRF between the TSA and UC arms (mean difference, −1.38 [95% CI, −2.36 to −0.41]; *P* = .002) in favor of the TSA arm; however, there was no statistically significant difference between the TSA and SSA arms (mean difference, −0.41 [95% CI, −1.41 to 0.58]; *P* = .96). There were no statistically significant differences between any of the arms at the week-12 visit. SSA was only statistically significant compared with UC at week 24 (mean difference, −0.97 [95% CI, −1.93 to −0.02]; *P* = .45).

The proportion of women who were no longer clinically fatigued (BFI <4) at week 6 was 58.5% (24 of 41) in the TSA arm, 51.1% (23 of 45) in the SSA arm, and 17.6% (9 of 51) in the UC arm. At week 24, 56.8% (21 of 37) in the TSA arm, 47.5% (19 of 40) in the SSA arm, and 21.7% (10 of 46) in the UC arm continued to have CRF, although the difference between arms was only statistically significant between the TSA arm and the UC arm at 6 weeks (TSA vs UC: OR, 6.59 [95% CI, 2.55-17.05]; *P* < .001) and 24 weeks (TSA vs UC: OR, 4.73 [95% CI, 1.82-12.29]; *P* < .001) (Table 1).

**Table 1.** Cancer Fatigue by Study Arm and Week

BFI analysis by arm	No.	Group mean difference (primary outcome)^a^				Proportion with BFI <4^b^		
		Mean (95% CI)		True vs sham, true vs usual care, adjusted mean difference (95% CI)	*P* value	No./total No. (%)	Odds ratio (95% CI)	*P* value
		Baseline	Follow-up					
Week 6^c^								
True	41	5.79 (5.28 to 6.30)	3.72 (3.15 to 4.28)	NA	NA	24/41 (58.5)	1 [Reference]	NA
Sham	45	5.38 (4.88 to 5.88)	4.04 (3.50 to 4.58)	−0.32 (−1.28 to 0.64)	>.99	23/45 (51.1)	1.35 (0.58 to 3.17)	.49
Usual care	51	5.38 (4.86 to 5.90)	4.94 (4.42 to 5.46)	−1.23 (−2.17 to −0.29)	.005	9/51 (17.6)	6.59 (2.55 to 17.05)	<.001
Week 12								
True	36	5.79 (5.28 to 6.30)	4.06 (3.47 to 4.65)	NA	NA	19/36 (52.8)	1 [Reference]	NA
Sham	44	5.38 (4.88 to 5.88)	4.31 (3.77 to 4.86)	−0.26 (−1.24 to 0.73)	>.99	21/44 (47.7)	1.22 (0.51 to 2.96)	.65
Usual care	48	5.38 (4.86 to 5.90)	4.79 (4.26 to 5.32)	−0.73 (−1.70 to 0.24)	.21	16/48 (33.3)	2.24 (0.92 to 5.43)	.08
Week 24								
True	37	5.79 (5.28 to 6.30)	3.69 (3.11 to 4.28)	NA	NA	21/37 (56.8)	1 [Reference]	NA
Sham	40	5.38 (4.88 to 5.88)	4.10 (3.54 to 4.67)	−0.41 (−1.41 to 0.58)	.96	19/40 (47.5)	1.45 (0.59 to 3.56)	.42
Usual care	46	5.38 (4.86 to 5.90)	5.07 (4.54 to 5.61)	−1.38 (−2.36 to −0.41)	.003	10/46 (21.7)	4.73 (1.82 to 12.29)	<.001

Abbreviations: BFI, Brief Fatigue Inventory; NA, not applicable.

^a^ Data for examination of group mean difference by study group were calculated using linear mixed regression models for all time points.

^b^ Scoring is based on 9 items, each scored on a 0-to-10 scale, with the final score calculated as the mean of completed items. Scores of 4 or more indicate clinically relevant cancer-related fatigue, 6 or more indicate severe cancer-related fatigue, and a 1.33-point reduction or a drop below 4 is considered clinically meaningful. Data for examination of odds ratios were calculated using logistic regression models. Odds ratios greater than 1 favor true acupressure.

^c^ Data were calculated for participants who had baseline and week-6 BFI scores. Primary end point.

### Secondary Aims

There were no statistically significant differences between the arms at any time point for sleep quality or for the proportion of participants who achieved normal sleep quality. For quality of life, more women had a clinically meaningful improvement in quality of life in the TSA arm (23 of 40 [57.5%]) compared with both the SSA (17 of 45 [37.8%]) and the UC (15 of 50 [30.0%]) arms at week 6, although this was only statistically significant between the TSA and UC arms (OR, 2.85 [95% CI, 1.20-6.80]; *P* = .02). Similarly, at week 24, the TSA arm (21 of 37 [56.8%]) had significantly more women reach meaningful improvements in quality of life vs the UC arm (15 of 45 [33.3%]; OR, 2.63 [95% CI, 1.07-6.45]; *P* = .04). The SSA arm had no significant difference in quality of life with the UC or the TSA arm at any time point (Table 2).

**Table 2.** Sleep Quality and Quality of Life by Study Arm and Week

Analysis by arm	No.	Group mean difference^a^				Proportion with PSQI ≤5 and FACT-O TOI with a ≥5-point increase^b^		
		Mean (95% CI)		True vs sham, true vs usual care, adjusted mean difference (95% CI)	*P* value	No./total No. (%)	Odds ratio (95% CI)	*P* value
		Baseline	Follow-up					
**PSQI global**								
Week 6								
True	53	9.32 (8.33 to 10.31)	8.26 (7.20 to 9.33)	NA	NA	11/40 (27.5)	1 [Reference]	NA
Sham	56	9.73 (8.77 to 10.70)	9.31 (8.28 to 10.33)	−1.04 (−2.85 to 0.77)	.50	9/45 (20.0)	1.52 (0.55 to 4.16)	.42
Usual care	51	8.59 (7.58 to 9.60)	8.67 (7.65 to 9.69)	−0.41 (−2.21 to 1.40)	>.99	7/49 (14.3)	2.28 (0.79 to 6.56)	.13
Week 12								
True	40	9.32 (8.33 to 10.31)	8.13 (7.03 to 9.23)	NA	NA	8/36 (22.2)	1 [Reference]	NA
Sham	45	9.73 (8.77 to 10.70)	8.62 (7.60 to 9.65)	−0.49 (−2.33 to 1.34)	>.99	12/44 (27.3)	0.76 (0.27 to 2.13)	.60
Usual care	49	8.59 (7.58 to 9.60)	8.74 (7.71 to 9.76)	−0.61 (−2.44 to 1.23)	>.99	7/49 (14.6)	1.67 (0.55 to 5.14)	.37
Week 24								
True	37	9.32 (8.33 to 10.31)	8.06 (6.97 to 9.15)	NA	NA	12/37 (32.4)	1 [Reference]	NA
Sham	40	9.73 (8.77 to 10.70)	8.58 (7.53 to 9.62)	−0.52 (−2.37 to 1.33)	>.99	11/40 (27.5)	1.27 (0.48 to 3.36)	.64
Usual care	45	8.59 (7.58 to 9.60)	8.16 (7.12 to 9.19)	−0.10 (−1.94 to 1.74)	>.99	10/45 (22.2)	1.68 (0.63 to 4.49)	.30
**FACT-O TOI**								
Week 6								
True	40	63.81 (60.24 to 67.38)	71.19 (67.37 to 75.01	NA	NA	22/40 (57.5)	1 [Reference]	NA
Sham	45	64.64 (61.17 to 68.12)	68.43 (64.77 to 72.09)	2.76 (−3.71 to 9.23)	.92	17/45 (37.8)	2.01 (0.85 to 4.79)	.11
Usual care	50	65.44 (61.80 to 69.08)	65.60 (61.94 to 69.26)	5.59 (−0.88 to 12.06)	.12	15/50 (30.0)	2.85 (1.20 to 6.80)	.02
Week 12								
True	36	63.81 (60.24 to 67.38)	70.03 (66.13 to 73.93)	NA	NA	19/36 (52.8)	1 [Reference]	NA
Sham	44	64.64 (61.17 to 68.12)	69.66 (65.98 to 73.33)	0.37 (−6.19 to 6.92)	>.99	24/44 (54.5)	0.93 (0.39 to 2.25)	.88
Usual care	48	65.44 (61.80 to 69.08)	66.85 (63.16 to 70.54)	3.18 (−3.39 to 9.74)	.73	17/48 (35.4)	2.04 (0.84 to 4.92)	.11
Week 24								
True	37	63.81(60.24 to 67.38)	70.76 (66.88 to 74.63)	NA	NA	20/37 (56.8)	1 [Reference]	NA
Sham	40	64.64 (61.17 to 68.12)	68.90 (65.16 to 72.64)	1.86 (−4.74 to 8.45)	>.99	20/40 (50.0)	1.31 (0.53 to 3.22)	.55
Usual care	45	65.44 (61.80 to 69.08)	66.24 (62.51 to 69.97)	4.52 (−2.06 to 11.10)	.30	15/45 (33.3)	2.63 (1.07 to 6.45)	.04

Abbreviations: FACT-O TOI, Functional Assessment of Cancer Therapy-Ovarian Trial Outcome Index; NA, not applicable; PSQI, Pittsburgh Sleep Quality Index.

^a^ Data for examination of group mean difference by study group were calculated using linear mixed regression models.

^b^ PSQI scoring is based on the sum of the 7 component scores, ranging from 0 to 21, with higher scores indicating worse sleep quality. FACT-O TOI scoring is based on a multidimensional 26-item summary score that includes the 27-item FACT-General core and 12 ovarian cancer-specific items; higher scores indicate a better quality of life, and a clinical meaningful difference is considered to be a change of 5 to 7 points. Data for examination of odds ratios were calculated using logistic regression models. Odds ratios greater than 1 favor true acupressure.

### Adherence, Fidelity, and Adverse Events

The mean (SD) adherence to the TSA protocol was 84% (47%), with a 2% to 177% range, which is equivalent to 22.7 minutes per day. In the SSA arm, the mean (SD) adherence was 78% (37%), and the range was 0% to 151%, or 21.1 minutes per day (*P* = .21 between acupressure arms). Mean (SD) fidelity to self-acupressure treatment was 88% (19%) in the TSA arm and 86% (16%) in the SSA arm with no significant difference between arms. There were no related serious or nonserious adverse events reported in any arms.

## Discussion

In this randomized clinical trial among ovarian cancer survivors, use of self-acupressure, as taught by a mobile app, resulted in a 58.5% decrease in CRF in the TSA arm compared with a 51.1% reduction in the SSA arm and a 17.6% reduction in the UC arm at the end of the 6-week intervention. Improvements in CRF were maintained in both self-acupressure arms at week 24. A clinically meaningful improvement in quality of life was also observed in the TSA arm compared with the UC arm. Despite improvements in quality of life and fatigue, no significant differences were seen in sleep quality.

In our team’s prior studies of self-acupressure for fatigue among cancer survivors, the acupressure technique was taught in person and applied using fingers, thumbs, or a pencil eraser.^13,14,31^ In the present study, we have demonstrated that self-acupressure can be taught effectively via a mobile app and applied using an acupressure device. Furthermore, adherence to self-acupressure was high, and reductions in CRF observed in the TSA arm were similar to those observed in prior studies (70%^14^ to 66%^13^), in which the technique was taught in person. Also, the treatment effect that we observed in the TSA arm was similar to the fatigue reductions seen with cognitive behavioral therapy, exercise, and mindfulness therapies, but critically, self-acupressure and the training via the app are free.^32,33,34^

Our prior research found that breast cancer survivors with CRF have alterations in brain neurochemistry within the posterior insula and disturbed functional connectivity to the default-mode network compared with breast cancer survivors without fatigue^11,35^ and that 6 weeks of TSA impacted functional connectivity to the default-mode network and posterior insula.^12^ These results suggest that TSA could be impacting CRF through these neural pathways. Further research into the biologic mechanism underlying the observed decrease in CRF in the current study among ovarian cancer survivors is warranted.

SSA had smaller but meaningful impacts on CRF, which could be due to a placebo effect. However, given that our sham acupoints were located in the same region of the body as our true acupoints and considering that acupressure impacts peripheral sensory nerves ultimately impacting central nervous system functioning,^32^ it is possible that the sham points also stimulated similar peripheral nerves to the TSA points. This could have led to similar impacts on CRF.

### Limitations

This study has limitations. Most of the women in this study were non-Hispanic White (86%), which limited our ability to generalize to other race and ethnicity groups. However, most women diagnosed with ovarian cancer in the US are non-Hispanic White (82.5%).^36^ In addition, more women withdrew from the self-acupressure arms: 29% in the TSA and 22% in the SSA compared with 7% in the UC arm, suggesting that self-acupressure is not for everyone; yet it offers a safe, inexpensive option for those who do participate.

## Conclusions

In this randomized clinical trial, self-acupressure, taught by an app, was found to be an inexpensive, safe, and effective therapy for improving CRF to normal fatigue levels in approximately 60% of women with ovarian cancer using TSA. Few trials, to our knowledge, that have investigated therapies for CRF have included people with ovarian cancer. Only 2 randomized clinical trials in the newest American Society of Clinical Oncology-Society for Integrative Oncology clinical guidelines included people with ovarian cancer (with a total of 216 ovarian cancer survivors).^32,33^ Of these 2 studies, only 1—a home-based cognitive behavioral therapy and exercise intervention—showed a statistically significant improvement in CRF.^32^ Given the large burden of CRF in this population, more research and therapies are needed.

## Notes

Supplement 1.
Trial Protocol

Supplement 2. **eTable. **Sociodemographic and Clinical Characteristics by Treatment Group **eFigure. **Location of True and Sham Self-Acupressure Points

Supplement 3.
Data Sharing Statement
