# Supplementary material for: Self-Acupressure for Fatigue in Patients Surviving Ovarian Cancer: A Randomized Clinical Trial
Source: JAMA Netw Open. 2026 Feb 5;9(2):e2556357. doi: 10.1001/jamanetworkopen.2025.56357 (PMC12878437; doi:10.1001/jamanetworkopen.2025.56357)
Supplement: Supplement 1. — Trial Protocol [file jamanetwopen-e2556357-s001.pdf]

Supplement 1. Protocol for Acupressure for Fatigue in Ovarian Cancer Survivors: AcuOva Study

Principal Investigator:

Suzanna M. Zick, ND, MPH

University of Michigan Medicine

Dept. of Family Medicine

Ann Arbor, MI

Co-Investigators:

Richard E Harris, PhD

UCI School of Medicine

Susan Samueli Integrative Health Institute

Irvine, CA

Ananda Sen, PhD

University of Michigan Medicine

Dept. of Family Medicine

Ann Arbor, MI

Celeste Leigh Pearce, PhD

University of Michigan Medicine

Dept. of Epidemiology

Ann Arbor, MI

Project Manager:

Sara Snyder, MBA

University of Michigan Medicine

Dept. of Family Medicine

Ann Arbor, MI

UM IRBMED #00143509:

UMCC 2018.130

Contents

1.0 BACKGROUND AND SIGNIFICANCE 4
Risk Assessment 7
Benefit Assessment 7
Hypotheses and Objectives 7
2.0 SPECIFIC AIMS 7
3.0 METHODS 8
Study Design. 8
3.1 Participants. 8
3.2 Recruitment. 8
3.3 Randomization 9
3.4 Blinding. 10
4.0 DESIGN CONSIDERATIONS. 11
4.1 Study Measures. 12
5.0 REPORTING ADVERSE EVENTS 16
5.1 Definition 16
5.2 Assessment of Relationship of AE to Treatment 16
5.3 Study Specific Adverse Event Reporting Plan 16
5.4 Follow-up of AEs 17
5.5 AE Reporting and Data Elements 17
5.6 Severity of AEs 17
6.0 CONCOMITANT MEDICATION, EXERCISE, DIET AND SMOKING 18
6.1 Limitations on Medication, Exercise, Diet and Smoking 18
6.2 Documentation of Medication 18
7.0 OFF-STUDY CRITERIA 18
7.1Study Termination 18
7.2 Premature Removal of a Participant 18
7.2.1 Personal reason 18
7.2.2 Lost to follow-up 19
7.2.3 Death 19
7.2.4. Lost to follow-up after enrollment but withdrew before the baseline visit 19
8.0 DATA MANAGEMENT 19
8.1 Statistics Section 19
9.0 STATISTICAL METHODS 20
9.1 Definition of an Evaluable Participant 21
9.2. Post-hoc Power Analysis 21
10.0 ETHICAL AND REGULATORY CONSIDERATIONS 23
10.1 Institutional Review Board (IRB) Approval 23
10.2 Informed Consent 23
10.3 Data and Safety Monitoring 23
10.3.1 Guidelines 23

10.3.2 Study-Specific DSMP 23
10.4 Record Retention 24
11.0 REFERENCES 25

1.0 BACKGROUND AND SIGNIFICANCE

Fatigue and Ovarian Cancer.

Women diagnosed with ovarian carcinoma (cancer) report multiple quality of life challenges, including persistent fatigue and sleep disturbances.[1-3] Persistent fatigue is a commonly reported symptom in these women, with as many as 30 to 60% of ovarian cancer survivors continuing to report significant fatigue even several years after the end of treatment.[1,4-7] In ovarian cancer, persistent fatigue has been linked to higher levels of chronic pain,[6,8] lower quality of life,[9,10] and reduced participation in other self-care activities such as physical activity.[11,12] Interestingly, risk factors for fatigue post cancer treatment does not appear to be associated with type of cancer, cancer treatment, stage of diagnosis or histopathology of the cancer, but instead with other host factors such as presence of depression or level of physical activity.[8,13] While the etiology of persistent fatigue in cancer survivors is unknown, recent research in female cancer survivors suggests that cancer related fatigue may involve pathology within the brain, and thus treatments that target the brain may have promise for treating fatigue.[14,15] Despite the challenges caused by persistent fatigue, there have been very limited attempts to identify self-care-focused interventions, which could improve fatigue, sleep, and quality of life among ovarian cancer survivors. We are proposing to conduct a randomized, controlled trial comparing self- administered acupressure to sham acupressure and no treatment to determine if this is an intervention that survivors can use to address their fatigue.

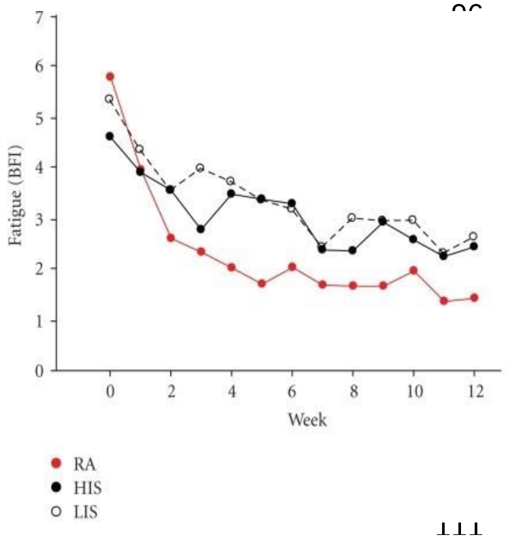

Figure 1 A plot of mean weekly fatigue scores for relaxing acupressure (red circles), high intensity stimulating acupressure (closed black circles), and low intensity stimulating acupressure (open black circles) across study weeks.

Acupressure and Fatigue

Self-administered acupressure is one possible safe, self- management technique for improving persistent fatigue as well as physical and psychosocial functioning in ovarian cancer survivors with chronic cancer-related fatigue. Acupressure, a technique derived from acupuncture, is a component of Traditional Chinese Medicine in which pressure is applied to specific acupoints on the body using a finger or small device to address health issues. Acupressure has shown promise for treating fatigue in cancer patients and survivors, although these have been small studies with numerous methodological flaws including inclusion of multimodal treatment approaches, patients currently receiving or recently completing cancer treatment, and no examination of carry-over effect of acupressure.[16-20] We performed a pilot intervention of relaxing and stimulating self-acupressure in fatigued adult cancer survivors, of which 14% had been diagnosed with ovarian cancer.[21] In this study, participants were randomized to one of three groups: 1. relaxing acupressure (n=14); 2. High intensity stimulating acupressure (n=15); or 3. low intensity stimulating acupressure (n=14). Participants performed acupressure for 12 weeks between 3 to 14 times per week depending on group. Fatigue was measured with the Brief Fatigue Inventory on a weekly basis. The change in the Brief Fatigue Inventory was significantly different across groups, with self-administered relaxing acupressure engendering greater reductions in fatigue when compared to acupressure. Deviation acupressure 2.2±1.6, low p=0.027). In a either high or low intensity stimulating (See Figures 1 and 2; mean ± standard reduction in Brief Fatigue Inventory: relaxing 4.0±1.5, high intensity stimulating acupressure intensity stimulating acupressure 2.7±2.2; linear regression model with change in Brief Fatigue Inventory as the dependent variable, the group difference remained significant after adjusting for age, cancer type, cancer stage, and cancer treatments (p=0.013). Across groups, these reductions in fatigue were about 45% to 70%, which are clinically relevant and could represent significant improvements in quality of life for cancer survivors.[21,22] **Acupressure was effective in reducing fatigue in other cancer survivors, including women with ovarian cancer.**

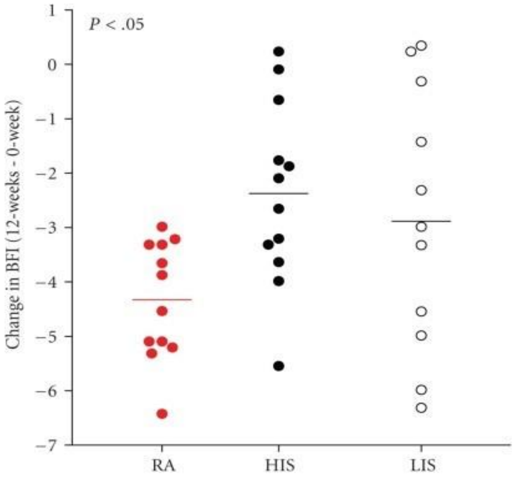

Figure 2 Scatter plot of individual participant BFI change scores (week 12— week 0) indicate greater reductions for relaxing acupressure (red circles), high intensity stimulating acupressure (closed black circles), and low intensity stimulating acupressure (open black circles).

As a demonstration that a larger study is feasible, we recently completed a randomized clinical trial in 288 fatigued breast cancer survivors (stage 0 to III), which demonstrated that self-administered acupressure was an effective intervention for persistent fatigue.[22] Survivors were randomized to one of two different types of self- acupressure (relaxing acupressure or stimulating acupressure) or usual care. After 6 weeks of daily treatment, both acupressure arms had significantly reduced fatigue compared to usual care and this effect persisted for one-month post-treatment (Figure 3). The mean percentage fatigue reductions at 6 weeks were 34%, 27%, and −1% in relaxing acupressure, stimulating acupressure, and usual care, respectively. Moreover, over 60% of women in the treatment arms achieved normal fatigue levels compared to only 31% in the usual care arm. Relaxing acupressure also significantly improved sleep quality and improved quality of life when compared to the usual care arm.[22] Further, acupressure worked equally well despite stage or histopathology of the cancer. Women were able to perform the self-acupressure with a high level of fidelity, i.e. the accuracy with which the acupressure treatment was performed by participants including correct location of acupoints, amount of pressure, duration and frequency of treatments (95% out of 100% on their fidelity ratings), and adherence (performed 73% of all acupressure sessions on average).[22,23] **These data indicate that acupressure was effective in reducing fatigue in female cancer survivors, and importantly, survivors can be recruited through the Michigan Cancer Registry and a large randomized, controlled study completed. However, no study has examined using a mobile app to teach female cancer survivors to self-administer acupressure — an important innovation to help increase implementation and accessibility for cancer survivors.**

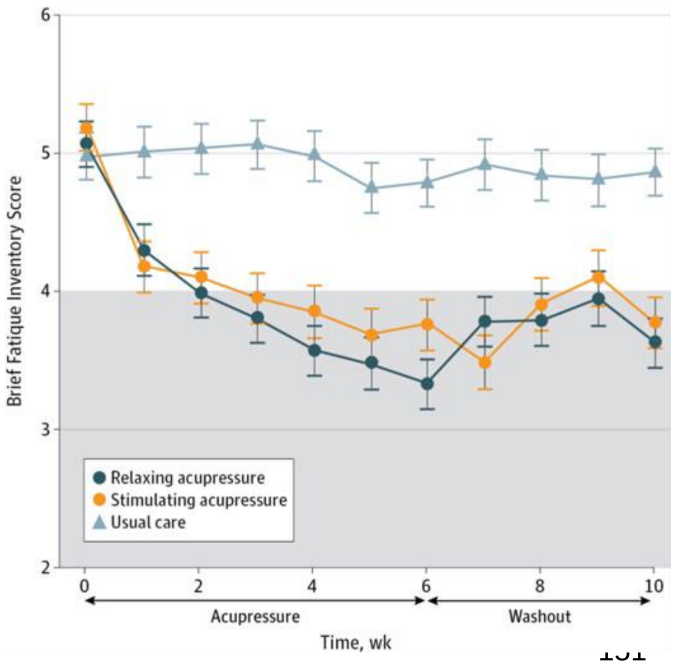

Figure 3 Fatigue by Week and Group Assignment. The Brief Fatigue Inventory consists of 9 items, each measuring fatigue on a scale of 0 to 10, and the score is calculated from the mean of completed items. Scores of 4 or higher indicate clinically relevant fatigue.

Self-Administered Acupressure

An advantage of self-acupressure is that once a person learns the technique, they are free to control the frequency and dose of the intervention. Since the intervention can be easily learned (generally in less than 15 minutes),[24] we reasoned that an online or mobile application (app) could increase its utilization. In a recent pilot survey of cancer patients with fatigue, use of technology such as a computer program or smartphone app was the most popular response for learning acupressure. Consequently, we developed a mobile app in association with focus groups consisting of cancer survivors (six focus groups consisting each of eight to ten women) and the University of Michigan 3D Media Laboratory. **The acupressure app is called MeTime Acupressure** (Figure 4). One concern with some app-based interventions is that the company that initially develops the app may cease to make it available or support it. **Our app was developed at the University of Michigan by the Principal Investigator** and thus there is no chance it will not be available in the future. Importantly, further refinements of the app can be done in an iterative manner.

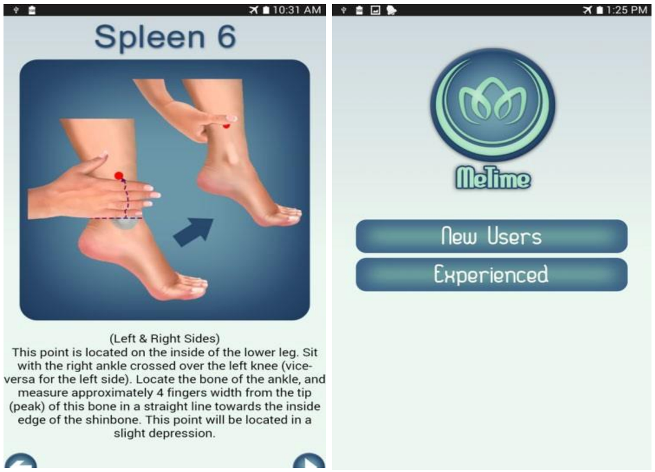

Figure 4 Sample MeTime Acupressure Screen

Focus group participants also asked for a device to accompany the app to learn and apply an accurate amount of pressure to each acupoint (the hardest skill to learn for participants and one they expressed as being of high importance).[23] They also wanted a device that was comfortable to use

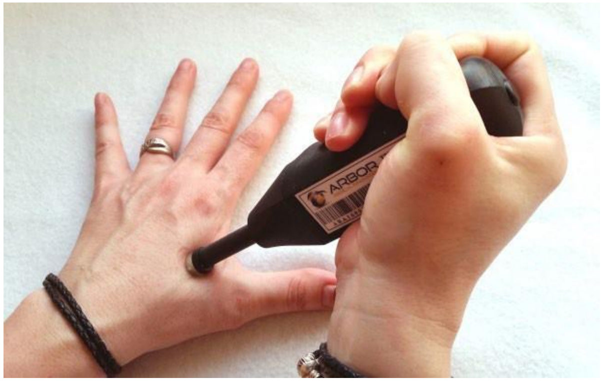

Figure 5 AcuWand prototype

and could minimize any strain in the hand or fingers from applying the acupressure. To achieve this goal, we partnered with Arbor Medical Innovations (AMI), a Michigan-based technology company to translate their expertise in handheld devices, which provide precise amounts of pressure to create the "AcuWand" device (see Figure 5). The AcuWand is a digital force gauge for the controlled delivery of pressure to discrete acupressure points throughout the body. The AcuWand applies forces from 0-100N, at 100ms intervals, to match the typical delivery of acupressure stimulation and gives feedback through visual and audio cues letting participants know when "too much" or "too little" pressure is being applied. It also incorporates an ergonomic handle so the user can easily grasp the device and apply pressure through a sprung probe with a soft rubber tip. **The AcuWand was designed in collaboration with users and can objectively and comfortably enable ovarian cancer survivors to deliver an adequate amount of pressure to each acupressure point – a critical aspect for performing reliable and efficacious acupressure treatment.**

Mechanism of Action of Relaxing Acupressure.

It is important to have a "biological rationale" for how stimulating certain areas of the body with a somatic stimulus, such as a finger applying pressure, could reduce fatigue any more so than beyond a simple placebo. For instance, a lack of a mechanism could impede the acceptance of, recommendation, and adoption of acupressure by medical professionals. While currently the underlying mechanisms explaining how acupressure improves fatigue and alleviates sleep disruption are unclear, recent research by our group and others provide important clues to systemic and central nervous system processes. A recent review by Zhao[25] suggested that fatigue and sleep effects of acupressure are mediated by a number of neurotransmitters including norepinephrine, melatonin, gamma-aminobutyric acid, and β-endorphin. Animal studies also indicate down-regulation of the hypothalamic–pituitary– adrenal axis, which reduces plasma corticosterone and adrenocorticotropin hormone levels.[26] A heterogeneous and large number of acupoints and methods of stimulating, e.g., needle insertion, electrical acupuncture, moxibustion, acupressure etc., were examined for their mechanistic effects. In general, the type of stimulation appears unimportant, while the acupoint location appears to have some impact on affecting these neuroendocrinological changes.[25] Given the importance of acupoint location it is germane to know that four out of the five acupoints we propose using in the relaxing acupressure formula are commonly used for insomnia in clinical trials:[25] heart 7; spleen 6; liver 3; and Animan, and have demonstrated effects on sleep-related neurotransmitter systems and hypothalamic–pituitary–adrenal axis activity in basic, human and animal studies.[26-36] Our own small-scale study, [15] which examined the effect of two different types of acupressure--relaxing and stimulating--on fatigue in breast cancer survivors using magnetic resonance imaging, also found that the different types of acupressure (distinguished only by their acupoint locations) work via different mechanisms within the brain. In particular, we found that the relaxing acupressure formula increased the connectivity between the superior colliculus – a brainstem region important in humans and primates that regulates sleep and alertness – and the default mode network – a network of brain regions that is activated when thinking about one's internal state. In comparison stimulating acupressure decreased this same connection (see Figure 6).[15] Moreover, for relaxing (but not stimulating) acupressure, these changes were associated with improvements in sleep quality.

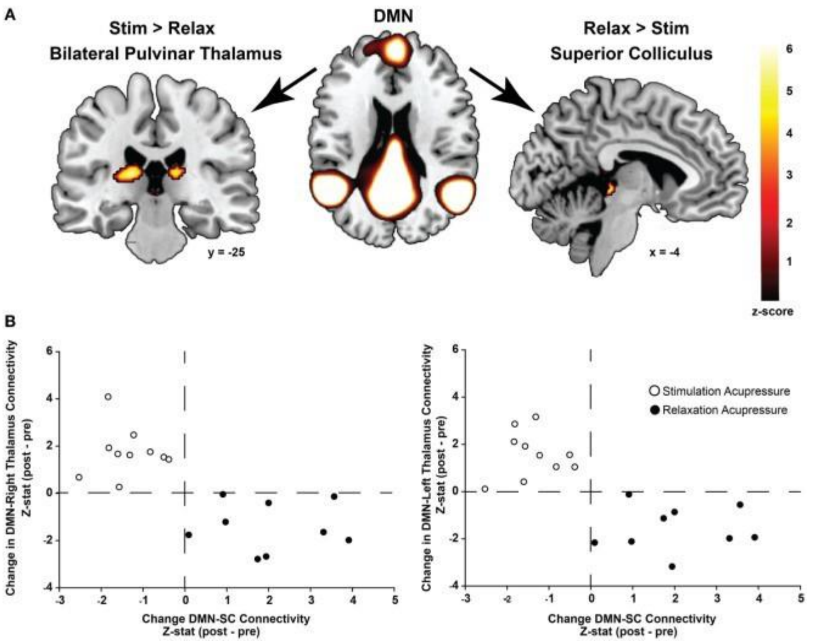

Figure 6 Differential changes in connectivity to the default mode network (DMN) following different acupressure treatments. (A) An independent component analysis of resting state functional MRI data shows increased connectivity between the to the superior colliculus (SC)/periaqueductal gray and the DMN following relaxation acupressure but not stimulating acupressure. Increased connectivity between the DMN and the thalamus (pulvinar) was observed following stimulating but not relaxing acupressure. (B) Scatter plots showing changes in DMN connectivity by acupressure treatment (stimulation: open circles, relaxation: black circles). Connectivity patterns are mutually exclusive: increases in SC-DMN connectivity are accompanied by decreases in pulvinar- DMN connectivity for relaxation acupressure, while the converse

Risk Assessment

The known or expected risks are risk of bruising at points of pressure; there is a very small chance (<1%) of having bruises at points of pressure if too much pressure is applied. This bruising should not continue or get worse. The researchers will try to minimize the risk of bruising by having the acupressure app demonstrate the correct amount of pressure to apply at the start of the study. Additionally, a member of the research team will call the participant at certain times during the study to evaluate their health and safety. During these conversations the study coordinator will ask the participant about any bruising.

Risk lowering measures: Study procedures to manage or minimize risks include a vibration feature installed in the AcuWand programming to signify or "warn" participants that minimum pressure has been achieved.

The AcuWand is not FDA approved, nor are we seeking FDA approval for its use. We do not anticipate any problems with the device which would cause injury or harm in any way.

Benefit Assessment

Participants may not receive any personal benefits from being in this study. However, it is possible that the acupressure treatments could reduce the severity of fatigue they are experiencing. We hope the information learned from this study will benefit other ovarian cancer patients.

Hypotheses and Objectives

We are proposing to evaluate the impact of **six weeks of daily self-administered relaxing acupressure** on fatigue, sleep, and quality of life in persistently fatigued ovarian cancer survivors compared to 6 weeks of daily self-administered sham acupressure or no treatment. Survivors who have fatigue starting at or after cancer diagnosis, and have ≥4 on the Brief Fatigue Inventory, which is the cutoff for moderate fatigue, will be eligible for the study.[37] During the study period we will randomize and follow 171 survivors (we anticipate a ~13 % dropout with the goal of 150 completers). We hypothesize that persistently fatigued ovarian cancer survivors will experience improvements in fatigue, sleep, quality of life, and symptoms commonly associated with persistent fatigue from utilizing relaxing as compared to sham acupressure or no treatment.

2.0 SPECIFIC AIMS

To investigate our hypothesis, we will randomize fatigued ovarian cancer survivors into 6 weeks of daily: 1. Relaxing self-acupressure (n=57); 2. Sham self-acupressure (n=57); or 3. No treatment (n=57) followed by 18 weeks of no acupressure and investigate the following aims:

1. Examine the effect of 6 weeks of daily self-administered relaxing acupressure compared to self-administered sham acupressure or no treatment on:

- **A.** Persistent fatigue (**Primary aim**) -- measured with The Brief Fatigue Inventory;
- **B.** Quality of life (**Secondary aim**) -- using the Functional Assessment of Chronic Illness Therapy Measurement System, specifically the ovarian cancer specific instrument;
- **C.** Sleep Quality (**Secondary aim**) -- using the Pittsburgh Sleep Quality Index;
- **D.** Symptoms commonly associated with persistent fatigue (**Exploratory aim**) (sleep disturbance, pain, cognitive function, mood, and female sexual function) -- using Patient Reported Outcomes Measurement Information System.

2 (Exploratory aim). To investigate the duration of effect self-administered relaxing acupressure as compared to self-administered sham acupressure or no treatment during the 18 weeks following the end of acupressure treatment (carry-over phase) on persistent fatigue, quality of life, sleep quality, and symptoms commonly associated with persistent fatigue (sleep disturbance, pain, cognitive function, mood, and female sexual function) using the measurement tools described for the primary aim.

Both acupressure treatments will be delivered via the MeTime Acupressure app in association with the AcuWand.

3.0 METHODS

Study Design.

We propose a randomized, controlled clinical trial comparing relaxing acupressure to sham acupressure and no treatment for persistent fatigue in ovarian cancer survivors. The study will be 25 weeks in duration: **Phase I** (**screening phase**; 7 days in duration/weeks -1 to 0), consisting of telephone screen for study eligibility, obtaining informed consent, random assignment to treatment group, and shipment of computer tablet and AcuWand, if appropriate; **Phase II** (**active treatment phase**; 6 weeks in duration), which will consist of performing once daily self-acupressure—sham or relaxing—with the study app and the AcuWand or no treatment with outcomes including baseline and 6-week subjective measures of fatigue; sleep quality, pain, female sexual function, cognition, and quality of life; **Phase III** (**carry-over phase**) is an 18-week follow-up to evaluate fatigue, sleep quality, pain, female sexual function, cognition, and quality of life after completion of the acupressure treatments with outcomes assessed at 12 and 24 weeks. Our studies have indicated that the effects of self- acupressure can persist for 4 weeks after the end of treatment and acupuncture interventions in chronic pain populations have seen sustained effects ranging from 6 months to one-year post-intervention.[38,39] As such, we intend to determine if acupressure has sustained impacts beyond 4 weeks after the end of treatment. **All groups including the no treatment group will have an equal number of study contacts.**

3.1 Participants.

Table 1 Eligibility Criteria

**Inclusion Criteria**	**Exclusion Criteria**
✓ Women aged 21 years and older.<br>✓ Diagnosis of ovarian cancer, stages I to IV.<br>✓ Have an average persistent fatigue defined as ≥ 4 on the Brief Fatigue Inventory.<br>✓ Fatigue must have started at or after the diagnosis of ovarian cancer.<br>✓ Initiated all primary cancer treatments. (surgery, chemotherapy at least 3 weeks prior), including maintenance therapies.<br>✓ Undergone any primary debulking surgery at least 4 weeks prior.<br>✓ Undergone interval debulking surgery at least 4 weeks prior if they have had neoadjuvant chemotherapy (NACT).<br>✓ No other planned interventions for fatigue other than current stable medication.	x Medically unstable.<br>x Acupressure or acupuncture receipt in the past year.<br>x Have a diagnosis of untreated mood disorder, e.g., bipolar or major depressive disorder.<br>x Have a current diagnosis of anemia.<br>x Have a current untreated diagnosis of hypothyroidism.<br>x Have an initiation, a cessation or change of treatment of any chronic medications, dietary supplements, behavioral therapy, physical therapy etc., or any planned change of medications, supplements or therapies during the study.<br>x Have the possibility of becoming pregnant.<br>x Diagnosis of COVID-19 on or after 3/1/2020 with fatigue onset following diagnosis

3.2 Recruitment.

Participants will be identified through a variety of sources. The Michigan Department of Health and Human Services (MDHHS) Michigan Tumor Registry collects information about cancer incidence, stage, and to a limited extent, the kinds of treatment that patients receive. These data are reported to the registry from various medical facilities. The Michigan Tumor Registry includes cancer case information received from the metropolitan Detroit Surveillance, Epidemiology, and End Results (SEER) registry. The Michigan Cancer Surveillance Program, which operates the Michigan Tumor Registry, is a member of the North American Association of Central Cancer Registries (NAACCR). The registry is certified by NAACCR as meeting all standards for quality, completeness, timeliness, and unresolved duplicate records and is estimated to be greater than 95% complete based on external audit findings.40,41 We will request Michigan Tumor Registry data for women diagnosed throughout the State of Michigan, even if they no longer reside in Michigan. Women will receive an introductory letter from the MDHHS describing the study and asking them to contact us if they are interested or if they would like to opt out of future mailings. A second letter will be sent to all women who do not respond within two weeks, and a final third letter to any non-responders from prior mailings two weeks after the second letter. Interested women from the Michigan database send their contact information to the MDHHS, who pass it along to the study team.

We will also be recruiting from the Los Angeles Cancer Surveillance Program (the Los Angeles SEER) and our local University of Michigan Cancer Registry in a similar manner. After approval from the State of California Cancer Registry IRB, they will provide contact information of potentially eligible women diagnosed with ovarian cancer and we will send them the same introductory letter being sent out by the MDHHS, a study flyer, and a brochure from the California Cancer Registry. A second letter will be sent to all

women from Los Angeles who do not respond within two weeks, and a final third letter to any non-responders from prior mailings two weeks after the second letter. Interested women from Los Angeles will be able to contact the study team directly. The study team then performs a phone screen to determine eligibility with all interested women. We will also contact a randomly selected group of women from the LA SEER registry directly via phone and/or text message (SMS).

We will also recruit from the North Carolina Central Cancer Registry (NC CCR) and follow the same process as LA SEER; we will receive contact information from the NC CCR for potentially eligible women and continue with our 3 communication letters or direct phone call and/or text message (SMS). If we don't receive any response from text messages from women from the NC CCR, we may send up to three letters via mail.

We will utilize the UM Cancer Registry to identify potentially eligible women diagnosed with ovarian cancer and will send them the same introductory and consecutive 2nd and 3rd/final recruitment letters. We may utilize certified mail for the final recruitment mailing for all participants contacted via mail. For participants identified through the UM Cancer Registry we may choose to contact them via phone or text message (SMS) for recruitment. We will also reach out to the leadership at ovarian cancer survivor groups, including, but not limited to the Michigan Ovarian Cancer Alliance (MIOCA), Ovarian Cancer Research Alliance (OCRA), Facing Our Risk of Cancer Empowered (FORCE), Michigan Oncology Quality Consortium (MOQC), and Ovarian Cancer Canada. We will email a study flyer and a short description of the study to the contact person at these at these organizations to allow them to distribute this information to their members as they deem appropriate. Interested women will again be able to contact the study team, who will perform the same phone screen.

Additionally, we will utilize Michigan Institute for Clinical and Health Research (MICHR) services to provide social media advertisement support. MICHR will manage all IRB approved advertising. We may also choose to use the images and texts in the MICHR advertisements to advertise through U-M Rogel Cancer Center Social Media and Health Communications Core.

We will work with the Michigan Department of Communications to assist us to recruit via paid advertisement on website search engines.

We will post the study on researchmatch.org. Like UMHealthResearch, Research Match is a participant recruitment and feasibility analysis tool for researchers at participating institutions, University of Michigan is a participating institution. We will post our study information here as well for potential participants to learn more and to enroll.

Social media accounts may be created to help recruit participants and guide them to our study. A detailed recruitment portfolio which includes imagery and text can be found in section 8-1.8 of our application.

We may choose to work with BuildClinical to help accelerate enrollment. BuildClinical is a health technology company that enables academic researchers to accelerate their clinical research patient recruitment efforts by engaging the exact population needed through digital advertising and ultimately reducing the patient access and awareness gaps that exist today.

We may choose to utilize the DataDirect resource at U-M to help identify potentially eligible participants. DataDirect is available for faculty researchers and their study team members to access both discrete variables and free-text string data from the electronic medical record (EMR).

Snowballing: We may choose to email participants who have completed study and share our flyer and social media content with them. If they would like to, they may share our study information to their own personal accounts or groups that they may be members of.

3.3 Randomization

The biostatistician will provide sealed envelopes for the randomization of all participants. Participants will be randomized to one of the three study arms: Acupressure, Sham acupressure or Control. A random generator for block stratified randomization in blocks of 6 participants will be programmed by Dr. Sen, the study statistician. Eligible patients will be randomized into one of three groups - relaxing acupressure, sham acupressure, or no treatment - in a 1:1:1 ratio for a total of 171 participants with 57 randomized into each group.

We will be delivering our self-acupressure interventions (relaxing and sham) using our modified MeTime Acupressure mobile app. We will rename the MeTime Acupressure app to "Study App" and change the icon so participants will not be able to locate it in the Apple

or Google Play Stores and unblind themselves. In association with the 3D Media Lab at the University of Michigan we will create a mobile app for our sham acupressure points. The sham and relaxing acupressure apps will be identical in all aspects except for the acupoint locations. For women randomized into the "no treatment arm" they will receive the acupressure app and AcuWand at the end of their study participation. The acupressure app will be loaded onto computer tablets by our computer information technology (IT) support person. Women in the acupressure arms will also receive an AcuWand to be used in association with the acupressure app to help women apply the correct amount of pressure to acupoints. The women will receive directions within the acupressure app on how to use the AcuWand. The AcuWand has been designed to use two lithium-ion long lasting (up to one year with normal use) batteries. Participants are asked to return the AcuWand with provided materials at no cost to them in order to upload data about frequency, duration and amount of pressure of acupressure applications. Of note: no Wi-Fi connection is needed to use the acupressure app and our IT support person will be available to help participants who are unfamiliar with or have any issues with their tablet, AcuWand or the app.

Relaxing Acupressure.

There are 5 acupoints with 4 of the acupoints performed on both the left and right sides of the body (total of 9 points to stimulate; see Figure 7). Each of the 9 acupoints will be stimulated for 3 minutes per point with the AcuWand giving a total treatment time of 27 minutes daily.

The relaxation acupoints are:

- *Yin tang* (Unilaterally): forehead, between eyebrows.
- *Anmian* (EX17) (Bilaterally): posterior aspect of the neck.
- Heart 7 (HT7) (Bilaterally): palmer surface of the hands on the wrist crease.
- Spleen 6 (SP6) (Right and Left/bilaterally): inside of the lower leg.
- Liver 3 (LV3) (Bilaterally): foot.

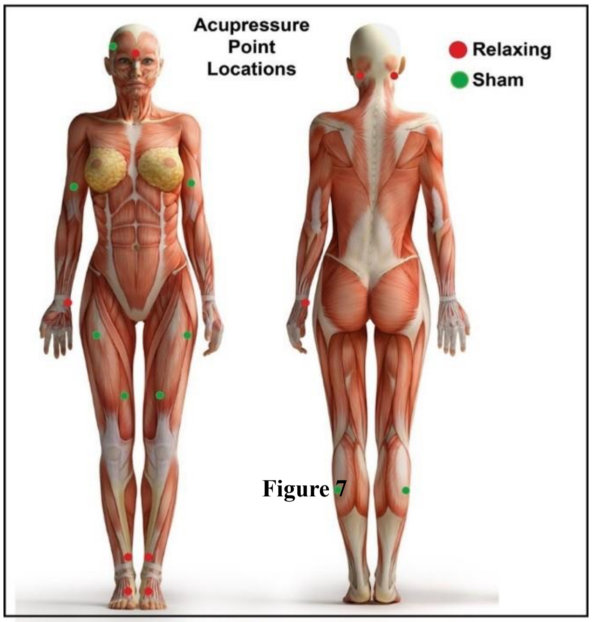

Figure 7

The relaxing points were selected based on existing data showing their effectiveness for treating fatigue.[21,22]

Sham Acupressure.

Sham acupoints were chosen by Dr. Harris in locations where no known acupoints exist. There are 5 acupoints with 4 of the acupoints performed on both the left and right sides of the body (total of 9 points to stimulate; see Figure 7). None of these points are on meridians nor are they near actual points (at least one inch away from meridians and points). They were chosen to be in the same general body quadrant as the relaxation points. Each of the 9 acupoints will be stimulated for 3 minutes per point giving a total treatment time of 27 minutes daily. The sham acupoints are:

- Pressure Point #1 (Unilaterally): This point is located on the side of the head, above the right ear.
- Pressure Point #2 (Bilaterally): This point is located on the muscle of the upper arm. Located in the middle point directly between the crease of the elbow (when arm is flexed) and the shoulder muscle one finger width toward the biceps muscle.
- Pressure Point #3 (Bilaterally): This point is located on the upper thigh muscle on the lateral side of the leg.
- Pressure Point #4 (Bilaterally): This point is located on the lower thigh muscle on the lateral side of the upper leg.
- Pressure Point #5 (Bilaterally): This point is located on the lateral side of the lower leg and is located midway between the line connecting the outer ankle bone and the knee.

3.4 Blinding.

Our IT support person will place the computer tablets—already loaded with either the acupressure app--sham or relaxing--or the "no treatment" message and the AcuWands into opaque shipping boxes per our randomization code, i.e., a tablet with the sham

acupressure app loaded on it would be placed in a shipping box labeled with a study ID assigned to the sham arm. The outside of the shipping boxes will be labeled with study IDs. This will ensure that only our IT support person will be unblinded, but all study staff and researchers will remain blinded until after the completion of data analyses. The IT support person will not be responsible for collecting or analyzing any study data. Participants in the “no treatment” arm will not be blinded; however, the two self-acupressure arms will be blinded as to which acupressure treatment they have been randomized. The MeTime app will be re-named to “Study App” for the study.

4.0 DESIGN CONSIDERATIONS.

Justification for Sham Acupressure and No Treatment Arms.

We chose to add a sham acupressure arm to this study so that we had a control arm that could be blinded to participants and study staff. Sham acupressure also controls for placebo (non-specific) effects associated with acupressure, and thus will allow us to estimate the specific or true effects of the relaxing acupressure treatment. The no treatment arm controls for regression to the mean and is an accurate reflection of what the majority of cancer survivors are receiving for their persistent fatigue from health care providers—no treatment.

Justification for Acupressure Dose (3 minutes per acupoints) and Duration (6 Weeks).

In our original acupressure pilot study in adult cancer patients,[21] we found that, regardless of treatment arm, the number of minutes performing acupressure was significantly correlated with reduction of fatigue or sleepiness. Participants (per their study logbooks) who stimulated their points for shorter periods of time, i.e., 1 minute, had less of an effect on fatigue and sleepiness compared to those who stimulated their points for 3 minutes. We also determined, from pilot work with fatigued cancer patients assigned to self-administered acupressure for 12 weeks, that change in fatigue improved over 6 weeks and then plateaued, with no further significant improvement.[21] Finally, our preliminary data from our randomized controlled trial in fatigued breast cancer subjects demonstrated improvements in subjective fatigue outcomes at 6 weeks. [22]

Justification for 24-Week Follow-up.

Our pilot data in fatigued adult breast cancer patients demonstrates that our 6-week acupressure intervention has sustained effects on fatigue, sleep and quality of life outcomes up to 4 weeks after the cessation of the acupressure treatment. Other acupuncture interventions mainly in chronic pain populations have seen sustained effects ranging from 6 months to one year post-intervention[39,42] and similarly, behavioral therapies for fatigue have sustained impacts of from 24 to 26 weeks (6 months).[43] For acupressure to be a widely used and implemented treatment we would need to observe sustained effects on fatigue reduction of the intervention similar to other pain treatments. As such, we are proposing a 24-week post-treatment follow- up for this study.

4.1 Study Measures.

Table 2 lists all study procedures and indicates with an ‘x’ during which visit a particular procedure is performed.

	Screening phone call	Baseline Week 0	Treatment Phase Weeks 1 - 6	Follow-up Week 6 (- 2 days/+5 days)	Follow-up Week 12 (- 2 days/+5 days)	Follow-up Week 24 (- 2 days/+5 days)
Phone Screen	X					
Screening Survey	X					
BFI	X	X		X	X	X
PHQ-2	X					
Consent	X					
Pt. Info & Dem.	X					
Concomitant of Medications		X				
Diagnosis & Treatment of initial cancer		X				
FACT-O		X		X	X	X
PSQI		X		X	X	X
PROMIS		X		X	X	X
IPAQ		X				
SCQ		X				
FSQ		X				
MAIA		X		X		
Daily Logbook			X			
Acupressure Fidelity				X		
Assessment of blinding				X		
AcuWand Comfort				X		
Final Evaluation						X

Administering Study Assessments.

All potential participants will be screened over the phone using a brief questionnaire to confirm eligibility for the study. Enrolled participants will then receive an email link to their RedCAP study measures at baseline, weeks 0, 6, 12 and 24. All the surveys will be administered at all-time points with the exception of descriptive and baseline assessments, which will only be collected at week 0; fidelity, blinding and adherence, which will only be asked at week 6; and a final evaluative questionnaire about the participants' perception of the study and the use of the AcuWand device. Adverse events will be collected at all-time points. Inspirational reminder emails, texts, or phone calls (as appropriate for a given participant) will be made to encourage the highest possible response rate to each round of study assessments. Participants without an email account will be assisted in obtaining a free Gmail account and how to access it on their computer tablet by study staff. Those participants who do not have access to Wi-Fi will be assisted in finding free Wi-Fi in their community (e.g., public library, Starbucks, McDonalds, etc.[44] IT study staff will be available to assist participants with any questions that may arise. E-mail is required so that study instruments may be accessed through surveys in RedCAP.

Screening. All potential participants will be screened over the phone using a basic questionnaire to confirm their eligibility. Specifically, an abbreviated form of the *Patient Health Questionnaire (PHQ-2)* will be used to detect untreated depression, and the *Brief Fatigue Index (below; BFI)* will be used to determine fatigue. These measures will be collected before consent and enrollment into the study and will therefore be kept to the absolute minimum to determine eligibility.

Rescreening. Participants may be rescreened at 6 weeks after initial screening if they failed their initial screening based on: stable chronic medications and no new planned interventions for their fatigue or cancer.

Fatigue Measure (administered at screening and weeks 0, 6, 12 ,24). *Brief Fatigue Inventory (BFI)*[37] will be used to assess the severity and impact of fatigue on daily functioning. The BFI is a scale validated in cancer patients with an alpha coefficient exceeding 0.95, which correlates well with other fatigue measures.[45] The BFI assesses severity and impact of fatigue in cancer patients over the past 24 hours. The instrument consists of 9 items, each measuring fatigue on a 0-10 scale, and is calculated from the mean of completed items. Scores of ≥4 indicate clinically- relevant fatigue.[37] A three-point change or a drop below 4 is considered a clinically meaningful change.[46]

Sleep Quality (administered weeks 0, 6, 12 ,24). *Pittsburgh Sleep Quality Index (PSQI)* is widely used 19-item questionnaire evaluates sleep disturbance over the past month. It yields a total score and 7 component scores: sleep quality; sleep latency; sleep duration; habitual sleep efficiency; sleep disturbances; use of sleep-promoting medication; and daytime dysfunction. In women with breast cancer, Cronbach's alpha for the global PSQI is 0.80.[47] A score >7 in breast cancer patients and thus likely in ovarian cancer patients suggests poor sleep quality.[48] The scale has adequate psychometrics.[49,50] The primary outcome will be the total PSQI score at baseline and week 6, although we will conduct exploratory analyses on the PSQI subscales.

Quality of Life (administered weeks 0, 6, 12 ,24). *The Functional Assessment of Cancer Therapy Ovarian (FACT-O)*[51] is a multidimensional questionnaire developed and validated for use by ovarian cancer patients; it includes the 27-item FACT-General (FACT-G) targeted to general cancer patients and 12 questions specific to issues faced by ovarian cancer patients (FACT-O subscale). The FACT-G questionnaire includes the following four subscales: physical well-being (PWB; seven items), social well-being (seven items), emotional well-being (six items) and functional well-being (FWB; seven items). These subscales can be analyzed separately or aggregated to produce a total health related quality of life score. The FACT-G has demonstrated reliability, validity and responsiveness to change over time. Two of the FACT-G subscales (PWB and FWB) plus the FACT- O subscale is summed to represent the total quality of life score.

Symptoms Associated with Fatigue (administered weeks 0, 6, 12 ,24). We will be using Patient Reported Outcomes Measurement Information System (PROMIS) measures. To diminish participant burden, we will be

using the short form versions of these measures. In a study in head and neck cancer patients a PROMIS battery asking about physical function, fatigue, sleep disturbance, depression, negative perceived cognitive function **took only 6.2 minutes to complete**.[52] The validity of PROMIS instruments has been assessed in both general and clinical U.S. sample populations including oncology populations.[53-55] PROMIS short forms represent the items of the PROMIS item banks constructed to measure the targeted constructs. All of the included PROMIS forms use 5-point Likert-type response categories to capture intensity, frequency, or duration, described in detail below. Most instruments use a seven-day recall period, with the exception of PROMIS Sexual Function and Satisfaction Profile which has a 30-day timeframe. PROMIS instruments are publicly available on the PROMIS Assessment Center Library website.[56] Following are detailed description of our included measures.

PROMIS Short Form v1.0 - Emotional Distress- Anxiety 7a[57] measures "fear, anxious misery, hyperarousal, and somatic symptoms related to arousal". The instrument consists of 7 items that ask respondents about the frequency with which they experienced emotions such as fear, stress, and anxiety ("never" to "always"). Scores range from 36.3 to 82.7 with higher scores indicating worse anxiety.

PROMIS Short Form v1.0 - Emotional Distress - Depression 8b[57] measures "negative mood, decrease in positive affect, information processing deficits, negative views of the self, and negative social cognition"[57]. It consists of 8 items in which respondents indicate the frequency with which they have experienced emotions such as worthlessness, hopelessness, and sadness ("never" to "always"). Scores range from 35.2 to 82.4 with higher scores representing worse depression.

PROMIS Short Form Sexual Function and Satisfaction Profile v1.0 – Female[58] - A collection of 11 brief (1-3) item short forms assessing interest in sexual activity, orgasm, global satisfaction with sex life, lubrication, and vaginal discomfort. The profile is intended for broad use, although almost all of the development work was with cancer populations. Scores range from 25.2 to 84.4 with higher scores representing worse sexual function.

PROMIS Short Form v1.0 - Sleep Disturbance 8b[59] This is an 8-item measure of perceptions of sleep quality, sleep depth, and restoration associated with sleep. Scores range from 28.9 to 76.5 with higher scores representing worse sleep disturbance.

PROMIS Short Form v1.0 - Pain Interference 8a[60] measures "impact of pain on physical, mental, and social activities". It consists of 8 items measured on a 5-point Likert-type scale (first 5 questions: "not at all" to "very much"; final question: "never" to "always"). The questions assess the degree to which pain interferes with enjoyment in life, ability to concentrate, day-to-day activities, recreational activities, tasks away from home, and socializing with others. The scores range from 41 to 78.3 with higher scores representing worse pain impact.

PROMIS Short Form v2.0 - Cognitive Function 8a[61] measures "mental acuity, concentration, verbal and nonverbal memory, verbal fluency, and perceived changes in these cognitive functions. The extent to which cognitive impairments interfere with daily functioning, whether other people observe cognitive impairments, and the impact of cognitive dysfunction on quality of life are also assessed. Scores range from 14.7 to 68.6 with higher scores representing worse cognitive function.

Multidimensional Assessment of Interoceptive Awareness (administered weeks 0, 6). *The Multidimensional Assessment of Interoceptive Awareness (MAIA)*[80] is a multidimensional questionnaire developed as a self-report measure of interoceptive body awareness. The MAIA includes an 8-scale state-trait questionnaire with 37 items to measure multiple dimensions of interoception by self-report. Participants score each answer on a range from 0 to 5 with total scores being calculated using the included rubric.

Descriptive/Baseline Variables (administered week 0).

Fibromyalgia Symptom Questionnaire (FSQ) consists of an assessment of widespread pain and symptom severity. The Widespread Pain Index (WPI) assesses the 19 specific body areas described in the survey criteria with scores ranging from 0-19. The second aspect of the criteria will be evaluated using the Symptom Severity (SS) scale (score

0-12). As per the survey criteria, patients will be classified as fibromyalgia positive (FM+) if their scores were WPI ≥ 7 and SS ≥ 5 or WPI = 3-6 and SS ≥ 9. The validity of the survey criteria has been established both when compared with the 1990 ACR criteria (which included the tender point examination) and the 2010 preliminary diagnostic criteria which include physician assessment.[78]

Concomitant Medications including dose, duration/frequency of use, and reason for use will be recorded. Sociodemographic Variables will include, but not be limited to: date of birth, race, ethnicity, marital status, education, and annual household income.

Cancer Diagnosis and Treatment. We will collect information about cancer treatments and diagnoses. These will be cross validated with data from the tumor registry.

Physical Activity. Because physical activity is known to impact the presence and severity of cancer-related fatigue and sleep disturbance[63,64] we will collect participants' activity using *The International Physical Activity Questionnaire (IPAQ).* The IPAQ assesses self-reported physical activity via a validated 7-day recall measure in which time and frequency spent performing activities of different intensities are recorded and tallied.[65,66]

Obesity and related comorbid conditions may also increase risk for common adverse cancer treatment effects, including fatigue, poor health-related quality of life, and worse functional health[67]. As such, we will be collecting:

Comorbidities using the *Self-Administered Comorbidity Questionnaire (SCQ).*[68,69] The SCQ is based on Katz's validated self-report adaptation of the Charlson Comorbidity Index to measure the presence and/or history of several chronic health conditions.

Body Mass Index (BMI) using a modified version of an e-health anthropometric questionnaire that includes questions dealing with self-measurement of current height, weight and has been found to highly correlate with in-person height and weight measurements.[70]

Safety, Fidelity, Blinding and Adherence (administered week 6 except for adverse events and adherence to acupressure, which are collected throughout the study).

Adverse Events will be graded according to National Cancer Institute Common Toxicity Criteria version 4.03.

Testing Participant Fidelity[21] We will be assessed using two methods. The first, as detailed in our prior study,[23] is done by research staff using a Skype face-to-face call (we will install Skype on the computer tablets and assist participants in using this program as needed.) Participants will be asked to identify the location, stimulation technique, and the amount of pressure (be able to demonstrate correct use of their AcuWand) of their acupoints. The number of acupoints correctly located will be recorded as will the adequacy of stimulating the acupoints (recorded as "yes" or "no"). The percentage of correct answers 0 to 100% will be calculated, based on circular (clockwise or counterclockwise) motion, maintaining contact with skin, and applying pressure such that the nail of the applying finger/thumb blanches. The second method will be data download from the AcuWand via Bluetooth when the computer tablet is connected to Wi-Fi. The AcuWand will be able to provide data on the duration, frequency, and amount of pressure used by participants-it cannot provide data on the location of where participants stimulated their points. We employ both methods to ensure that fidelity is measured across all parameters and in collect redundant measure in case an AcuWand does not transmit data.

Blinding will be assessed by asking participants, which type of acupressure they thought they were performing.

Adherence to Acupressure will be assessed by asking participants to record if and when they performed acupressure, the order in which they performed their acupressure points, and the amounts of achiness and/or tenderness they experienced while performing the acupressure on a Visual Analog Scale daily in the study

logbooks. These will be used in association with data downloaded from the AcuWands. Bi-weekly emails will be used to troubleshoot and encourage adherence.

<u>AcuWand Comfort Questionnaire (administered week 6).</u>

Massachusetts General Hospital Acupuncture Sensation Scale (MASS) consists of twelve predefined descriptors and one subjective component specified by the subjects in their own words to measure levels of *de qi* sensations.

De qi is a prerequisite term in Traditional Chinese Medicine (TCM) used to describe sensory stimulation. It has historically been thought to be an indicator of appropriate acupuncture or acupressure treatment and thus relates to therapeutic effectiveness.[79]

<u>Comfort level and ease of use</u> of the AcuWand device will also be assessed.

5.0 REPORTING ADVERSE EVENTS

5.1 Definition

An adverse event (AE) is any condition which appears or worsens after a participant is enrolled in an investigational study. An AE does not necessarily have a causal relationship with the study agent.

5.2 Assessment of Relationship of AE to Treatment

The possibility that the adverse event is related to study treatment will be classified as one of the following: not related, unlikely, possible, probable, definite.

5.3 Study Specific Adverse Event Reporting Plan

All Serious Adverse Events that are deemed related, probably related or possibly related will be reported to the IRBMED within 7 days, as per UM IRBMED guidelines.

All other non-serious, non-life-threatening adverse events will be reviewed by the PI and reported as per the following.

Study Specific Adverse Event Reporting Plan:

**Reportable Events**	**Timing of Report to IRBMED**
Unanticipated / unexpected problem involving risks to subjects or others	Serious - within 7 days<br><br>Non-Serious - with scheduled continuation review
Any physical, social, or psychological harm attributable to participation in this research study (e.g. an injury occurring during a study visit, bruising at the site of acupressure treatment)	Serious - within 7 days<br><br>Non-Serious - with scheduled continuation review
Death while on study	Within 7 days of notification
Loss of job or insurability due to breach or revelation of research records or participation.	Within 7 days of notification
**Non-Reportable Events**	

- Hospitalizations and morbidity expected in population (e.g. surgery for removal of fibroid tumor)
- Other serious or non-serious events deemed *not related* or *unlikely related* to the research

Serious = an event requiring hospitalization, permanent disability, incarceration, significant familial disruption (e.g. separation, divorce), job loss

Non-Serious = an event requiring some medical, psychological, psychiatric or similar attention to resolve.

Scheduled continuation review = renewal application.

5.4 Follow-up of AEs

All AEs will be followed according to good medical practices and documented as such.

5.5 AE Reporting and Data Elements

All participants will be questioned regarding adverse events during the follow up phone call and final visit. All participants will be instructed to contact the study coordinator via phone call or email at any time during the study if they have a concern.

All adverse events that occur after the informed consent is signed will be recorded on the adverse event case report form (CRF) whether or not related to study intervention.

The following information will be collected for all adverse events:

- AE reported date
- AE Verbatim Term
- CTCAE Term (v 4.03)
- Event onset date and event ended date
- Severity grade
- Attribution to study treatment (relatedness)
- Whether or not the event was reported as a Serious Adverse Event (SAE)
- Action taken with the study intervention
- Outcome of the event
- Comments

5.6 Severity of AEs

Severity will be graded according to NCI Common Terminology Criteria for Adverse Events (CTCAE) version 4.03. The CTCAE provides descriptive terminology and a grading scale for each adverse event listed. A copy of the CTCAE can be found at [http://ctep.cancer.gov].

AEs will be assessed according to the CTCAE grade associated with the AE term. AEs that do not have a corresponding CTCAE term will be assessed according to their impact on the participant's ability to perform daily activities as follows:

Grade	Severity	Description
1	Mild	• Asymptomatic or mild symptoms<br>• Clinical or diagnostic observations only<br>• Intervention not needed

2	Moderate	• Minimal, local or non-invasive intervention indicated<br>• Limiting age-appropriate instrumental activities of daily living
3	Severe	• Medically significant but not immediately life-threatening<br>• hospitalization or prolongation of hospitalization<br>• Disabling<br>• Limiting self-care activities of daily living
4	Life threatening	• Life threatening consequences<br>• Urgent intervention needed
5	Death	• Death related to Adverse Event

6.0 CONCOMITANT MEDICATION, EXERCISE, DIET AND SMOKING

6.1 Limitations on Medication, Exercise, Diet and Smoking

Concomitant prescribed medications are allowed except as listed in Exclusion Criteria. Acute medication will be allowed, defined as taking a medication for < 10 days over the course of a month with the intent to discontinue that medication.

Similarly, participants will be asked (once consented) to maintain their customary exercise, dietary and smoking habits during the screening, intervention and follow-up phase of the study. Participants will be asked to refrain from starting or stopping any new lifestyle modifications for the entire duration of the study.

6.2 Documentation of Medication

All chronic medications (prescription or OTC used for at least 14 days continuously), dietary supplements and/or herbal preparations taken by the participant during the study period will be documented on a CRF with information including:

- Type of medication
- Medication dosage/schedule
- Purpose for taking the medication, supplements or herbs (if available)

7.0 OFF-STUDY CRITERIA

7.1Study Termination

The study participant will be considered off-study when they complete the full study, as defined as completing the 24-week visit.

The Principal Investigator can decide to terminate a participant's participation in the study at any time. This decision could be based on factors such as unacceptable adverse events or for safety concerns. The reason for termination shall be documented in the case report form.

7.2 Premature Removal of a Participant

7.2.1 Personal reason

A participant may withdraw from the study at any time; the reason will be documented in patient study records.

7.2.2 Lost to follow-up

Three attempts must be made by telephone and letter to determine the circumstances for loss to follow-up, since such loss may be related to the study intervention.

7.2.3 Death

Participants who die while on study will be replaced by another participant.

7.2.4. Lost to follow-up after enrollment but withdrew before the baseline visit

Participants who withdraw between enrollment and the baseline visit can be replaced with a new participant

8.0 DATA MANAGEMENT

Study measures will be delivered electronically to participants using RedCAP. RedCAP is a generalized survey service supported by the University of Michigan Medical School permitting the creation and distribution of surveys, as well as data storage and analysis. RedCAP is user-friendly but can handle complex designs. For instance, simple surveys can be produced in minutes. Complex studies, involving randomization and embedded and longitudinal data, are also possible. RedCAP is also HIPAA compliant. RedCAP surveys will be accessed by the participant through an electronic device, via e-mail link forwarded from the study team, and are separate from the acupressure app. Those unable to effectively access and use these instruments will have a study team member administer these measures via phone, entered directly into RedCAP.

8.1 Statistics Section

The primary aim of this study is to investigate the differential effects of different modes of acupressure (relaxing vs. sham vs. no treatment) on fatigue as measured by the BFI in ovarian cancer survivors at week 6. The BFI, which measures the average degree of fatigue, gives a score that ranges between 0 and 10, with higher numbers indicating more fatigue. To investigate the difference in BFI between the three study arms, we shall use linear mixed-effects regression analysis with BFI as outcome, *week* as the within-subjects factor with four levels (0, 6, 12, 24) and *group* (relaxing acupressure, sham acupressure, no treatment) as the primary between-subjects factor. Of interest is the group-by-week interaction (to be included in the model), significance of which will indicate differences in extent of decrease between the arms. The covariate cancer stage at diagnosis and the interaction term group-by-stage will be added to models. Post-hoc sliced main effect of weeks within each study arm will be estimated. A *subject* random-effect will be used to account for the clustering effect within the four measurements on the same subject. Model diagnostics will be carried out to confirm the distributional assumptions and appropriate corrective actions (e.g. transformation) will be performed as needed. Models with more flexible covariance pattern (e.g. unstructured covariance, heterogeneity across groups) will be explored. An additional analysis with a spline version of weeks will be carried out which uses week as a continuous variable allowing one to estimate the rate of change in BFI.

A secondary analysis with BFI will also be carried out which investigates the difference in proportion of fatigued subjects (BFI $\geq$ 4) across the three study arms. The analytical framework will be that of a mixed-effects logistic regression model with presence of fatigue (BFI $\geq$ 4 yes/no) being the dichotomous outcome. Apart from group, week, and group-by-week interaction as primary covariates, the model will also be adjusted for baseline status of severity. Remaining covariates in the model are identical to those in the continuous BFI model. The clustering effect due to subject will be accounted for using a generalized estimating equations approach under a generalized linear mixed models framework.

Secondary Aims. Analogous linear mixed-effects regression analyses as described in the primary outcome analysis will be employed to investigate our secondary outcomes at week 6 the **Secondary Aim "B"**, FACT-O, of which we will use two of the Functional Assessment of Cancer Therapy general subscales (PWB and FWB) plus the FACT-O subscale summed to represent our quality of life outcome; and **Secondary Aim "C"**; PSQI, which has scores for seven components, namely sleep quality; sleep latency; sleep duration; habitual sleep

efficiency; sleep disturbances; use of sleep-promoting medication; and daytime dysfunction, all derived from the original PSQI. The component scores are summed to obtain a total score, the outcome for the proposed study.

Since PSQI score greater than 5 is considered an indicator of poor sleep quality, we shall also carry out a logistic regression analysis with a dichotomous outcome indicating whether PSQI > 5 is satisfied or not. This will compare the proportions (longitudinally) of subjects with poor sleep quality across the study arms. The analytical framework will be very similar to that for testing equality of proportions of subjects that are fatigued.

Exploratory Aims. Analogous linear mixed-effects regression analyses as described in the primary outcome analysis will be employed to investigate our exploratory outcomes (all administered on weeks 0, 6, 12, and 24) the summation of the PROMIS Short Form questions covering: anxiety, depression, pain interference, sleep disturbance, cognitive function, and sexual function and satisfaction profile.

To investigate the duration of effect of self-administered relaxing acupressure as compared to self-administered sham acupressure or no treatment during the 18 weeks following the end of acupressure treatment (carry-over phase) on persistent fatigue, quality of life, sleep quality, and symptoms commonly associated with persistent fatigue (sleep disturbance, pain, cognitive function, mood, and female sexual function) we will break up the observation period into two phases. In the first phase BFI and other study measures will be obtained by averaging week 0-week 6 measures and will indicate the status until end of the acupressure treatment (treatment-phase). The second phase comprises of week 7-week 24 and indicates the washout phase (washout). To investigate the difference in BFI and other study measures between the three study arms, we shall use mixed- effects regression analysis with average BFI and other study measures in the two phases as outcome, phase as the within-subjects factor and group (RA, SA, standard of care) as the primary between-subjects factor. We shall carry out an analysis with the phase variables as treatment-phase and washout as the levels. The purpose of investigating the model will compare the differences between the treatment and washout period across groups (phase-group interaction).

Handling Missing Data. The *linear mixed-effects* framework is an intent-to-treat framework in the sense that it uses all available data at all time-points. So if there are missing outcome values in any phase due to participant dropout, the usual analysis will still be valid. If the proportion of dropouts is substantial (20% or more) in either arm, then we shall also perform a weighted analysis with weights equal to the inverse of the probability of dropout. On the other hand, missing covariate values for the subject-level information will be imputed using multiple imputation methods. All missing values will be imputed using the chained equation method.[72] The advantage of using this technique is its flexibility in allowing different types of variables (categorical and continuous) to be imputed together without requiring any multivariate joint distributional assumption. In this method, the missing values are sequentially updated using bootstrap or Markov Chain Monte Carlo based on multiple regression models with other variables as covariates. This procedure will be carried out for a number of repetitions or cycles, thereby constructing an 'imputed' dataset. Ten such 'imputed' datasets will be used for the final analysis, a number which is considered adequate for most applications. Finally, we shall combine the results from the ten regressions with the imputed data using Rubin's formula.[73]

9.0 STATISTICAL METHODS

Power Analysis. Our power analysis is based on our primary aim to, "Examine the effect of 6 weeks of daily self-administered relaxing acupressure compared to self-administered sham acupressure or no treatment on persistent fatigue as measured with The Brief Fatigue Inventory." Although we will run a larger model for the analysis, in the power calculation we focus on the specific components of the model in which we are interested. Towards that end, we compute the power via simulation using a mixed effects model with a between-subject factor group (3 levels), a within-subject factor week (0, 6, 12, and 24), group-by-time interaction and a random subject effect. The mean BFI at baseline is assumed to be 5.5, whereas values at end of treatment were assumed to be 4.5, 3.5, and 2, respectively for no treatment, sham acupressure and relaxing acupressure arms. The between-subject variance is assumed to be 4 at all time-points whereas the variance of the random subject component is taken to be 4 also (yielding an intra-class correlation of 0.5). These assumed values are estimates based on our pilot data.[21] For this configuration, the power for detecting a significant week-by-group interaction is greater than 95%

with a sample size of 50 evaluable participants per treatment arm (we anticipate a ~13 % drop out with the goal of 150 completers for a total sample size of 171) and a 5% level of significance. Such a model is able to detect a post-hoc difference between the relaxing acupressure and each of the other two arms with more than 90% power at a reduced 0.01 level of significance in order to protect against Type I error probability inflation. In the second phase after the treatment period, it is anticipated that both the no-treatment and the sham acupressure arms will experience a bounce back close to the baseline level, while the relaxing acupressure arm will maintain its end-of-treatment average. Thus, the differences will be more pronounced than those in the active treatment phase, and we will be well powered to detect the effect size.

We also want to power the study to allow us to observe differences in sleep parameters. The extent of overlap between fatigue and sleep disturbances in ovarian cancer survivors is currently unknown. Research indicates that anywhere from 55 to 67% of ovarian cancer survivors experience sleep disruptions.[7,74] While fatigued women are most likely enriched with individuals experiencing sleep disturbances we will conservatively assume that roughly 50% of women will have some significant sleep disturbances per treatment arm. As such, with 50 women per treatment group we will be overpowered for detecting a difference in fatigue and sufficiently powered to detect changes in sleep measures. For example, using the PSQI as a basis for powering differences in sleep quality from previous studies the mean PSQI in female cancer survivors ranges from 6.84 □ 0.376 to 7.16 □ 0.325.[47] A 1-point decrease in the PSQI from ~7 to ~6 is considered a clinically significant difference. The mean PSQI values at baseline is assumed to be 7 in all arms whereas the means are taken to be 7, 6, and 5 at 6 weeks in the no treatment, sham and relaxing acupressure arms, respectively. Assuming an intra-subject correlation of 0.5, we have powers above 95% to detect differences between any pair of arms at 1% level of significance.

All power calculations are carried out on the basis of 100 simulations in the software PASS 2008 (NCSS, Kaysville, Utah 84037, USA).

9.1 Definition of an Evaluable Participant

Evaluable participants must complete both the baseline and week 6 BFI.

9.2. Post-hoc Power Analysis

Average changes in BFI between baseline and 6 weeks for both true and sham acupressure were statistically significant at 5% level, so power was not an issue in detecting these effect sizes. It is evident from Table 4 that although it did not reach statistical significance, the difference between true and sham acupressure was trending towards that (p = 0.08). It turns out that with the mean of the changes estimated to be that obtained in the data (Table 3), in order to detect a difference between the two Acupressure arms with 80% power, we would have needed 94 subjects per arm. The calculation is based on a two-sample t-test with a pooled standard deviation estimate of 1.7.

Table 3 Mean and standard deviation of changes between baseline and 6-weeks by study arm.

	N	Mean	Std. Deviation
True Acupressure	41	-1.9621	2.04466
Sham Acupressure	45	-1.2988	1.63485
Usual Care	51	-.4336	1.51849
Total	137	-1.1752	1.82757

Table 4

Multiple Comparisons

Dependent Variable: Change in BFI between Baseline and week 6 visit

LSD

(I) Arm	(J) Arm	Mean Difference (I-J)	Std. Error	Sig.	95% Confidence Interval	
					Lower Bound	Upper Bound
True Acupressure	Sham Acupressure	-.66329	.37307	.078	-1.4012	.0746
	Usual Care	-1.52851*	.36246	.000	-2.2454	-.8116
Sham Acupressure	True Acupressure	.66329	.37307	.078	-.0746	1.4012
	Usual Care	-.86521*	.35341	.016	-1.5642	-.1662
Usual Care	True Acupressure	1.52851*	.36246	.000	.8116	2.2454
	Sham Acupressure	.86521*	.35341	.016	.1662	1.5642

*. The mean difference is significant at the 0.05 level.

Study Limitations and Challenges.

As with any study we anticipate potential barriers**.** Recruitment may prove to be one potential challenge; however, our methods of recruiting breast cancer survivors from the Michigan Tumor Registry[41] have been effective and we are planning similar methods in this study. We can also recruit from the members of the Michigan Ovarian Cancer Science and Innovation Consortium of which Dr. Pearce is a member. Attrition from the study may be another challenge. We have had experience in keeping participants in fatigue and cancer studies[21,22,75] with higher levels of study burden compared to this proposal for at least 3 months. In our previous studies, which included four in-person visits, twice as many questionnaires, and offered no participant incentives such as a free computer tablet, our withdrawal rates ranged from 10% to 20%.[21,22] As such, we have planned for a 10% withdrawal rate for this proposal given our lower level of participant burden and greater study incentives.

We also understand that not all women are familiar with or use computers, mobile apps, Wi-Fi, or email. This may be a particular concern for older women who will make up a significant portion of the proposed study's participant pool as ovarian cancer rates are highest in women aged 55-64 years with the median age at which women are diagnosed being 63.[76] The most recent PEW statistics on internet and technology shows that roughly half of all Americans own tablet computers and this has been swiftly rising (see Figure 8). Computer tablet ownership is higher in younger, more educated, white urban adults. Despite this, 37% of adults aged 55-65 and 32% 65+ own a tablet.[77] To address those women who are unfamiliar or have limited familiarity with computer tablets we will provide support from a dedicated research IT personnel Mr. Budde. Mr. Budde has extensive experience offering computer support to a wide range of computer users of diverse skill sets and sociodemographic backgrounds. We will also supplement Mr. Budde's support with simple written directions geared to a seventh-grade level of education.

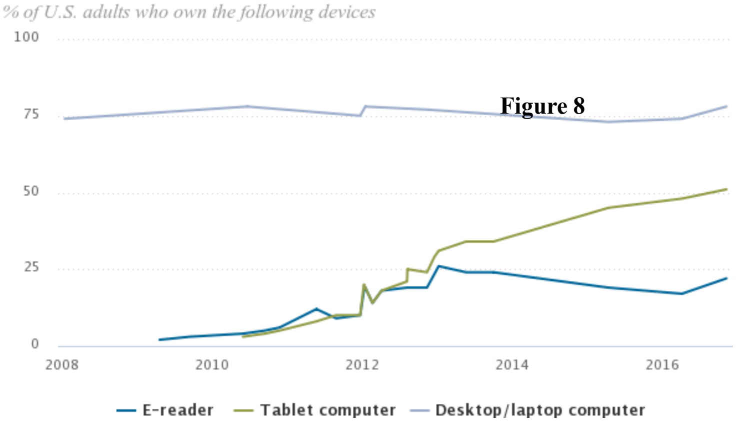

Figure 8

Conclusion

This proposal aims to investigate a low-cost, evidence-supported intervention for improving fatigue, sleep quality and quality of life in ovarian cancer survivors with persistent cancer-related fatigue. This patient- centered self-management intervention has the potential to be easily disseminated in healthcare settings and directly to the community via a mobile app. It could also be an adjunct to current treatment options for persistent cancer-related fatigue.

10.0 ETHICAL AND REGULATORY CONSIDERATIONS

10.1 Institutional Review Board (IRB) Approval

Prior to initiating the study, the PI must obtain written approval to conduct the study from the appropriate IRB. Should changes to study protocol become necessary, protocol amendments will be submitted electronically by the PI or designated study staff to the IRB for approval prior to implementation.

10.2 Informed Consent

All potential candidates for the study will be given an electronic copy of the study informed consent to read. The investigator or their designee will explain all aspects of the study in lay language and answer all the candidate's questions regarding the study. If the candidate decides to participate in the study, they will be asked to sign the informed consent document electronically. Participants who refuse to participate or who withdraw from the study will be treated without prejudice.

The informed consent document must be reviewed and approved by the IRB prior to study initiation. Any subsequent changes to the informed consent must be approved by the IRB for approval prior to implementation.

10.3 Data and Safety Monitoring

10.3.1 Guidelines

The purpose of the data and safety monitoring plan is to ensure the safety of participants, the validity of data, and the appropriate termination of studies for which significant benefits or risks have been uncovered or when it appears that the trial cannot be concluded successfully. Risks associated with participation in research must be minimized to the extent practical, and the method and degree of monitoring should be commensurate with risk. The essential elements of the Data and Safety Monitoring Plan include:

- Monitoring the progress of trials and the safety of participants
- Plans for assuring compliance with requirements regarding the reporting of adverse events (AE)
- Plans for assuring data accuracy and protocol compliance.

The known or expected risks associated with this study include:

- Risk of bruising at points of pressure; there is a very small chance (<1%) of having bruises at points of pressure if too much pressure is applied. This bruising should not continue or get worse. To minimize the risk of bruising study staff members will demonstrate the correct amount of pressure to apply at the start of the study. Additionally, a member of the research team will call participants during the study to evaluate their health and safety. During these conversations study team members will ask about any bruising.
- Risk of breach in confidentiality; there is a possible risk of the loss of confidentiality of participant medical information. The study team will try to protect participant privacy and the confidentiality of information.

10.3.2 Study-Specific DSMP

This study will be monitored in accordance with the NCI approved University of Michigan Rogel Cancer Center Data and Safety Monitoring Plan.

The study team will meet every six months or more frequently depending on the activity of the protocol. The discussion will include matters related to the safety of study participants (SAE/UaP reporting), validity and integrity of the data, enrollment rate relative to expectations, characteristics of participants, retention of participants, adherence to the protocol (potential or real protocol deviations) and data completeness. At these regular meetings, the protocol specific Data and Safety Monitoring Report form will be completed and signed by the Principal Investigator or by one of the co-investigators.

Data and Safety Monitoring Reports will be submitted to the University of Michigan Rogel Cancer Center Data and Safety Monitoring Committee (DSMC) every six months for independent review.

10.4 Record Retention

Clinical records for all participants studied, including CRFs, history and physical findings, laboratory data, and results of consultations, will be maintained by the Investigator in a secure storage facility and stored for at least five years after the study is closed.

11.0 REFERENCES

1. Holzner B, Kemmler G, Meraner V, et al. Fatigue in ovarian carcinoma patients: a neglected issue? *Cancer.* 2003;97(6):1564-1572.
2. Zhou Y, Irwin ML, Ferrucci LM, et al. Health-related quality of life in ovarian cancer survivors: Results from the American Cancer Society's Study of Cancer Survivors - I. *Gynecologic oncology.* 2016;141(3):543-549.
3. Clevenger L, Schrepf A, Degeest K, et al. Sleep disturbance, distress, and quality of life in ovarian cancer patients during the first year after diagnosis. *Cancer.* 2013;119(17):3234-3241.
4. Sekse RJ, Hufthammer KO, Vika ME. Fatigue and quality of life in women treated for various typesof gynaecological cancers: a cross-sectional study. *Journal of clinical nursing.* 2015;24(3-4):546-555.
5. Liavaag AH, Dorum A, Fossa SD, Trope C, Dahl AA. Controlled study of fatigue, quality of life, and somatic and mental morbidity in epithelial ovarian cancer survivors: how lucky are the lucky ones? *Journal of clinical oncology: official journal of the American Society of Clinical Oncology.* 2007;25(15):2049-2056.
6. Stavraka C, Ford A, Ghaem-Maghami S, et al. A study of symptoms described by ovarian cancer survivors. *Gynecologic oncology.* 2012;125(1):59-64.
7. Westin SN, Sun CC, Tung CS, et al. Survivors of gynecologic malignancies: impact of treatment on health and well-being. *Journal of cancer survivorship: research and practice.* 2016;10(2):261-270.
8. Shinde S, Wanger T, Novotny P, Grudem M, Jatoi A. Disease-free ovarian cancer patients report severe pain and fatigue over time: prospective quality of life assessment in a consecutive series. *European journal of gynaecological oncology.* 2015;36(2):155-160.
9. Ploos van Amstel FK, van Ham MA, Peters EJ, Prins JB, Ottevanger PB. Self-reported distress in patients with ovarian cancer: is it related to disease status? *International journal of gynecological cancer: official journal of the International Gynecological Cancer Society.* 2015;25(2):229-235.
10. Teng FF, Kalloger SE, Brotto L, McAlpine JN. Determinants of quality of life in ovarian cancer survivors: a pilot study. *Journal of obstetrics and gynecology Canada : JOGC = Journal d'obstetrique et gynecologie du Canada : JOGC.* 2014;36(8):708-715.
11. Stevinson C, Steed H, Faught W, et al. Physical activity in ovarian cancer survivors: associations with fatigue, sleep, and psychosocial functioning. *International journal of gynecological cancer: official journal of the International Gynecological Cancer Society.* 2009;19(1):73-78.
12. Stevinson C, Faught W, Steed H, et al. Associations between physical activity and quality of life in ovarian cancer survivors. *Gynecologic oncology.* 2007;106(1):244-250.
13. Bower JE. Cancer-related fatigue--mechanisms, risk factors, and treatments. *Nature reviews Clinical oncology.* 2014;11(10):597-609.
14. Zick SM, Zwickey H, Wood L, et al. Preliminary differences in peripheral immune markers and brain metabolites between fatigued and non-fatigued breast cancer survivors: a pilot study. *Brain imaging and behavior.* 2014;8(4):506-516.
15. Harris RE, Ichesco E, Cummiford C, et al. Brain Connectivity Patterns Dissociate Action of Specific Acupressure Treatments in Fatigued Breast Cancer Survivors. *Front Neurol.* 2017; 8:298.
16. Lan SC, Lin YE, Chen SC, Lin YF, Wang YJ. Effects of acupressure on fatigue and depression in hepatocellular carcinoma patients treated with transcatheter arterial chemoembolization: a quasi- experimental study. *Evidence-based complementary and alternative medicine: eCAM.* 2015; 2015:496485.
17. Molassiotis A, Sylt P, Diggins H. The management of cancer-related fatigue after chemotherapy with acupuncture and acupressure: a randomised controlled trial. *Complementary therapies in medicine.* 2007;15(4):228-237.
18. Tang WR, Chen WJ, Yu CT, et al. Effects of acupressure on fatigue of lung cancer patients undergoing chemotherapy: an experimental pilot study. *Complementary therapies in medicine.* 2014;22(4):581-591.
19. Zick SM, Alrawi S, Merel G, et al. Relaxation acupressure reduces persistent cancer-related fatigue.

Evidence-based complementary and alternative medicine: eCAM. 2011;2011(Article ID 142913):10.

20. Xu J, Gu L, Qiao H. Effect of Traditional Chinese and Western Medicine Joint Exercise Therapy on Cancer-Related Fatigue after Operation of Mammary Cancer. *Zhong Guo Quan Ke Yi Xue.* 2010; 13:3225-3226.
21. Zick SM. Relaxation Acupressure Reduces Persistent Cancer-Related Fatigue. *Evidence-based complementary and alternative medicine.* 2011;165(2):1-11.
22. Zick SM, Sen A, Wyatt GK, Murphy SL, Arnedt JT, Harris RE. Investigation of 2 Types of Self- administered Acupressure for Persistent Cancer-Related Fatigue in Breast Cancer Survivors: A Randomized Clinical Trial. *JAMA oncology.* 2016.
23. Wyatt GK, Frambes DA, Harris RE, Arnedt JT, Murphy SL, Zick SM. Self-administered Acupressure for Persistent Cancer-related Fatigue: Fidelity Considerations. *Altern Ther Health Med.*2015;21(4):18-23.
24. Balneaves LG, Lee RT, Guns ES, Zick SM, Bauer-Wu S, Greenlee H. Tenth International Conference of the Society for Integrative Oncology Translational Science in Integrative Oncology: from bedside to bench to best practices. *Integrative cancer therapies.* 2014;13(1):5-11.
25. Zhao K. Acupuncture for the treatment of insomnia. *International review of neurobiology.* 2013; 111:217- 234.
26. Park HJ, Park HJ, Chae Y, Kim JW, Lee H, Chung JH. Effect of acupuncture on hypothalamic-pituitary- adrenal system in maternal separation rats. *Cellular and molecular neurobiology.* 2011;31(8):1123- 1127.
27. Li M, Hu L, Cai RL, Wu ZJ, Wang KM. [Effects of electroacupuncture at PC6 and BL15 on nerve electrical activity in spinal dorsal root and norepinephrine and dopamine contents in paraventricular nucleus of hypothalamus in rats with acute myocardial ischemia]. *Zhong xi yi jie he xue bao = Journal of Chinese integrative medicine.* 2012;10(8):874-879.
28. Wang KM, Liu J, Wu ZJ, et al. [Relatively specific effect of electroacupuncture of different acupoints on hypothalamic monoamine neurotransmitters in myocardial ischemia rats]. *Zhen ci yan jiu = Acupuncture research / [Zhongguo yi xue ke xue yuan Yi xue qing bao yan jiu suo bian ji].* 2011;36(3):205-208, inside back cover.
29. Zhou Y, Wang Y, Fang Z, et al. [Influence of acupuncture on blood pressure, contents of NE, DA and 5- HT of SHR and the interrelation between blood pressure and whole blood viscosity]. *Zhen ci yan jiu = Acupuncture research / [Zhongguo yi xue ke xue yuan Yi xue qing bao yan jiu suo bian ji].* 1995;20(3):55-61.
30. Reynolds CF, 3rd. Troubled Sleep, troubled minds, and DSM-5. *Archives of general psychiatry.* 2011;68(10):990-991.
31. Lee BH, Zhao RJ, Moon JY, et al. Differential involvement of GABA system in mediating behavioral and neurochemical effect of acupuncture in ethanol-withdrawn rats. *Neuroscience letters.* 2008;443(3):213- 217.
32. Yoon SS, Kim H, Choi KH, et al. Acupuncture suppresses morphine self-administration through the GABA receptors. *Brain research bulletin.* 2010;81(6):625-630.
33. Zhou YL, Gao XY, Wang PY, Ren S. [Effect of acupuncture at different acupoints on expression of hypothalamic GABA and GABA(A) receptor proteins in insomnia rats]. *Zhen ci yan jiu = Acupuncture research / [Zhongguo yi xue ke xue yuan Yi xue qing bao yan jiu suo bian ji].* 2012;37(4):302-307.
34. Nordio M, Romanelli F. Efficacy of wrists overnight compression (HT 7 point) on insomniacs: possible role of melatonin? *Minerva medica.* 2008;99(6):539-547.
35. Cheng CH, Yi PL, Lin JG, Chang FC. Endogenous opiates in the nucleus tractus solitarius mediate electroacupuncture-induced sleep activities in rats. *Evidence-based complementary and alternative medicine: eCAM.* 2011; 2011:159209.
36. Cheng CH, Yi PL, Chang HH, Tsai YF, Chang FC. Kappa-opioid receptors in the caudal nucleus tractus solitarius mediate 100 hz electroacupuncture-induced sleep activities in rats. *Evidence-based complementary and alternative medicine: eCAM.* 2012; 2012:715024.
37. Mendoza TR, Wang XS, Cleeland CS, et al. The rapid assessment of fatigue severity in cancer patients: use of the Brief Fatigue Inventory. *Cancer.* 1999;85(5):1186-1196.
38. Manheimer E, Cheng K, Linde K, et al. Acupuncture for peripheral joint osteoarthritis.

Cochrane Database of Systematic Reviews. 2010;1:CD001977.

39. Yuan J, Purepong N, Kerr DP, Park J, Bradbury I, McDonough S. Effectiveness of acupuncture for low back pain: a systematic review. *Spine.* 2008;33(23): E887-900.

40. Meliker JR, Goovaerts P, Jacquez GM, Avruskin GA, Copeland G. Breast and prostate cancer survival in Michigan: can geographic analyses assist in understanding racial disparities? *Cancer.* 2009;115(10):2212-2221.

41. Zick SM, Wyatt GK, Murphy SL, Arnedt JT, Sen A, Harris RE. Acupressure for persistent cancer-related fatigue in breast cancer survivors (AcuCrft): a study protocol for a randomized controlled trial. *BMC complementary and alternative medicine.* 2012;12(1):132.

42. Manheimer E, Cheng K, Linde K, et al. Acupuncture for peripheral joint osteoarthritis. *Cochrane Database of Systematic Reviews.* 2010(Jan 5, 2010).

43. Heckler CE, Garland SN, Peoples AR, et al. Cognitive behavioral therapy for insomnia, but not armodafinil, improves fatigue in cancer survivors with insomnia: a randomized placebo-controlled trial. *Supportive care in cancer: official journal of the Multinational Association of Supportive Care in Cancer.* 2016;24(5):2059-2066.

44. Carlon K. Here's How to Get Free WI-FI Anywhere. 2017; https://www.androidpit.com/how-to-get-free- wi-fi-anywhere. Accessed 8-18, 2017.

45. Minton O, Stone P. A systematic review of the scales used for the measurement of cancer-related fatigue (CRF). *Annals of oncology: official journal of the European Society for Medical Oncology / ESMO.* 2009;20(1):17-25.

46. Vickers AJ, Straus DJ, Fearon B, Cassileth BR. Acupuncture for postchemotherapy fatigue: a phaseII study. *Journal of clinical oncology: official journal of the American Society of Clinical Oncology.* 2004;22(9):1731-1735.

47. Berger AM, Kuhn BR, Farr LA, et al. Behavioral therapy intervention trial to improve sleep quality and cancer-related fatigue. *Psycho-oncology.* 2009;18(6):634-646.

48. Carpenter JS, Elam JL, Ridner SH, Carney PH, Cherry GJ, Cucullu HL. Sleep, fatigue, and depressive symptoms in breast cancer survivors and matched healthy women experiencing hot flashes. *Oncology nursing forum.* 2004;31(3):591-5598.

49. Krupp LB, LaRocca NG, Muir-Nash J, Steinberg AD. The fatigue severity scale. Application to patients with multiple sclerosis and systemic lupus erythematosus. *Arch Neurol.* 1989;46(10):1121-1123.

50. Carpenter JS, Andrykowski MA. Psychometric evaluation of the Pittsburgh Sleep Quality Index. *Journal of psychosomatic research.* 1998;45(1):5-13.

51. Cella DF, Tulsky DS, Gray G, et al. The Functional Assessment of Cancer Therapy scale: development and validation of the general measure. *Journal of clinical oncology: official journal of the American Society of Clinical Oncology.* 1993;11(3):570-579.

52. Stachler RJ, Schultz LR, Nerenz D, Yaremchuk KL. PROMIS evaluation for head and neck cancer patients: a comprehensive quality-of-life outcomes assessment tool. *The Laryngoscope.* 2014;124(6):1368-1376.

53. Cella D, Riley W, Stone A, et al. Initial Adult Health Item Banks and First Wave Testing of the Patient- Reported Outcomes Measurement Information System (PROMIS (™)) Network: 2005–2008. *Journal of clinical epidemiology.* 2010;63(11):1179-1194.

54. Pilkonis PA, Choi SW, Reise SP, Stover AM, Riley WT, Cella D. Item Banks for Measuring Emotional Distress from the Patient-Reported Outcomes Measurement Information System (PROMIS®): Depression, Anxiety, and Anger. *Assessment.* 2011;18(3):263-283.

55. Garcia SF, Cella D, Clauser SB, et al. Standardizing patient-reported outcomes assessment in cancer clinical trials: a patient-reported outcomes measurement information system initiative. *Journal of clinical oncology: official journal of the American Society of Clinical Oncology.* 2007;25(32):5106-5112.

56. Measures H. PROMIS (Patient Reported Outcomes Measurement Information System. 2017; http://www.healthmeasures.net/explore-measurement-systems/promis. Accessed 8-17,2017.

57. Cella D, Riley W, Stone A, et al. The Patient-Reported Outcomes Measurement Information System (PROMIS) developed and tested its first wave of adult self-reported health outcome item banks: 2005- 2008. *Journal of clinical epidemiology.* 2010;63(11):1179-1194.

58. Flynn KE, Reeve BB, Lin L, Cyranowski JM, Bruner DW, Weinfurt KP. Construct validity of the PROMIS® sexual function and satisfaction measures in patients with cancer. *Health and Quality ofLife Outcomes.* 2013;11(1):40.

59. Buysse DJ, Yu L, Moul DE, et al. Development and validation of patient-reported outcome measuresfor sleep disturbance and sleep-related impairments. *Sleep.* 2010;33(6):781-792.

60. Khanna D, Krishnan E, Dewitt EM, Khanna PP, Spiegel B, Hays RD. The future of measuring patient- reported outcomes in rheumatology: Patient-Reported Outcomes Measurement Information System (PROMIS). *Arthritis care & research.* 2011;63 Suppl 11: S486-490.

61. Cella D, Schalet BD, Lai JS, et al. PROSETTA STONE® ANALYSIS REPORT A ROSETTA STONE FOR PATIENT REPORTED OUTCOMES PROMIS V2.0 COGNITIVE FUNCTION AND FACT-COG PERCEIVED COGNITIVE IMPAIRMENT. 2014; J.http://www.prosettastone.org/LinkingTables1/Linking%20Tables%20vol2/PROMIS%20Cognitive%20F unction%20and%20FACT-Cog%20Perceived%20Cognitive%20Impairment%20Full%20Report.pdf. Accessed 8-18, 2017.

62. Spitzer RL, Kroenke K, Williams JB. Validation and utility of a self-report version of PRIME-MD: the PHQ primary care study. Primary Care Evaluation of Mental Disorders. Patient Health Questionnaire. *JAMA: the journal of the American Medical Association.* 1999;282(18):1737-1744.

63. Tomlinson D, Diorio C, Beyene J, Sung L. Effect of exercise on cancer-related fatigue: a meta-analysis. *American journal of physical medicine & rehabilitation / Association of Academic Physiatrists.* 2014;93(8):675-686.

64. van Vulpen JK, Peeters PH, Velthuis MJ, van der Wall E, May AM. Effects of physical exercise during adjuvant breast cancer treatment on physical and psychosocial dimensions of cancer-related fatigue: A meta-analysis. *Maturitas.* 2016; 85:104-111.

65. Craig CL, Marshall AL, Sjostrom M, et al. International physical activity questionnaire: 12-country reliability and validity. *Medicine and science in sports and exercise.* 2003;35(8):1381-1395.

66. Booth M. Assessment of physical activity: an international perspective. *Research quarterly for exercise and sport.* 2000;71 Suppl 2:114-120.

67. Schmitz KH, Neuhouser ML, Agurs-Collins T, et al. Impact of obesity on cancer survivorship and the potential relevance of race and ethnicity. *Journal of the National Cancer Institute.* 2013;105(18):1344- 1354.

68. Sangha O, Stucki G, Liang MH, Fossel AH, Katz JN. The Self-Administered Comorbidity Questionnaire: a new method to assess comorbidity for clinical and health services research. *Arthritis and rheumatism.* 2003;49(2):156-163.

69. Katz DA, McHorney CA. The relationship between insomnia and health-related quality of life in patients with chronic illness. *J Fam Pract.* 2002;51(3):229-235.

70. Lassale C, Peneau S, Touvier M, et al. Validity of web-based self-reported weight and height: results of the Nutrinet-Sante study. *Journal of medical Internet research.* 2013;15(8): e152.

71. Weimer E. Qualtrics. 2017; https://wiki.med.umich.edu/display/UMHSHELPDESK/Qualtrics. Accessed 8-25, 2017.

72. van Buuren S, Boshuizen HC, Knook DL. Multiple imputation of missing blood pressure covariates in survival analysis. *Stat Med.* 1999;18(6):681-694.

73. Little R, Rubin D. *Statistical analysis with missing data.* New York, New York: Wiley; 1987.

74. Sandadi S, Frasure HE, Broderick MJ, Waggoner SE, Miller JA, von Gruenigen VE. The effect of sleep disturbance on quality of life in women with ovarian cancer. *Gynecologic oncology.* 2011;123(2):351- 355.

75. Harris RE, Tian X, Williams DA, et al. Treatment of fibromyalgia with formula acupuncture: investigation of needle placement, needle stimulation, and treatment frequency. *Journal of alternative and complementary medicine (New York, NY).* 2005;11(4):663-671.

76. Alliance: OCRF. Cancer Stat Facts: Ovarian Cancer. 2015; https://ocrfa.org/patients/about-ovarian- cancer/statistics/. Accessed 8-18, 2017.

77. Anderson M. Technology Device Ownership: 2015. 2015; http://www.pewinternet.org/2015/10/29/technology-device-ownership-2015. Accessed 8-18, 2017.

78. Wolfe F, Clauw DJ, Fitzcharles MA, et al. Fibromyalgia criteria and severity scales for clinical and epidemiological studies: a modification of the ACR Preliminary Diagnostic Criteria for Fibromyalgia. J Rheumatol. 2011;38(6):1113-1122.
79. Jang JC, Jung J, Lee H, Park YB, Kim H. Multidimensional aspects of *de qi* sensations in MASS and ASQ assessment: a pilot study. *Evid Based Complement Alternat Med*. 2017;2017:6249329
80. Mehling WE, Price C, Daubenmier JJ, Acree M, Bartmess E, Stewart A. The Multidimensional Assessment of Interoceptive Awareness (MAIA). PLOS ONE 7(11): e48230.
