# Supplementary material for: Self-Acupressure for Fatigue in Patients Surviving Ovarian Cancer: A Randomized Clinical Trial
Source: JAMA Netw Open. 2026 Feb 5;9(2):e2556357. doi: 10.1001/jamanetworkopen.2025.56357 (PMC12878437; doi:10.1001/jamanetworkopen.2025.56357)
Supplement: Supplement 2. — eTable. Sociodemographic and Clinical Characteristics by Treatment Group eFigure. Location of True and Sham Self-Acupressure Points [file jamanetwopen-e2556357-s002.pdf]

Supplemental Online Content

Zick SM, Chen D, Harris RE, et al. Self-acupressure for fatigue in patients surviving ovarian cancer: a randomized clinical trial. *JAMA Netw Open*. 2026;9(2):e2556357.
doi:10.1001/jamanetworkopen.2025.56357

eTable. Sociodemographic and Clinical Characteristics by Treatment Group
eFigure. Location of True and Sham Self-Acupressure Points

This supplemental material has been provided by the authors to give readers additional information about their work.

© 2026 Zick SM et al. *JAMA Network Open*.

eTable. Sociodemographic and Clinical Characteristics by Treatment Group

	All N=171	True N=58	Sham N=58	Usual Care N=55
Age, Mean ± STD	56±12	57± 10	55± 12	57± 13
BMI, Mean ± STD	31± 9	29± 8	32± 8	32± 10
Race, N(%)				
Asian	4(2)	2(3)	0(0)	2(4)
Black or African American	4(2)	2(3)	1(2)	1(2)
Hispanic[a]	10(6)	1(2)	5(9)	4(7)
Native Hawaiian or other Pacific Islander	2(1)	0(0)	1(2)	1(2)
White non-Hispanic	147(86)	51(88)	51(88)	45(82)
Two or more races	1(0)	0(0)	0(0)	1(2)
Unknown	3(2)	2(3)	0(0)	1(2)
Cancer Stage, N(%)				
Stage I	48(28)	11(19)	16(28)	21(38)
Stage II	19(11)	11(19)	5(9)	3(5)
Stage III	62(36)	23(40)	19(33)	20(36)
Stage IV	19(11)	4(7)	10(17)	5(9)
Other	4(2)	0(0)	4(7)	0(0)
Unknown	19(11)	9(16)	4(7)	6(11)
Prior Surgery, N(%)				
Yes	121(71)	43(74)	43(74)	35(66)
Unknown	19(11)	6(10)	5(9)	8(15)
Chemotherapy, N(%)				
Currently Receiving	15(9)	5(9)	4(7)	6(11)
Completed	128(75)	42(72)	46(79)	46(84)
Unknown	28(16)	11(19)	8(14)	3(5)
Date of Initial Diagnosis, Months, Mean ± STD	48± 41	47± 44	49± 46	46±34
Type of Ovarian Cancer, N(%)				
High-grade serous	45(36)	14(34)	15(33)	16(41)
Low-grade serous	13(10)	1(2)	7(15)	5(13)
Endometrioid	24(19)	10(24)	6(13)	8(21)
Clear cell	9(7)	3(7)	5(11)	1(3)
Mucinous	5(4)	1(2)	2(4)	2(5)
Mixed-cell	3(2)	2(5)	1(2)	0(0)
Other	27(24)	10(24)	10(22)	7(18)
Unknown	45(26)	17(29)	12(21)	16(29)

a. Subjects that indicated Hispanic and non-White or unknown race.

© 2026 Zick SM et al. *JAMA Network Open*.

eFigure. Location of True and Sham Self-Acupressure Points

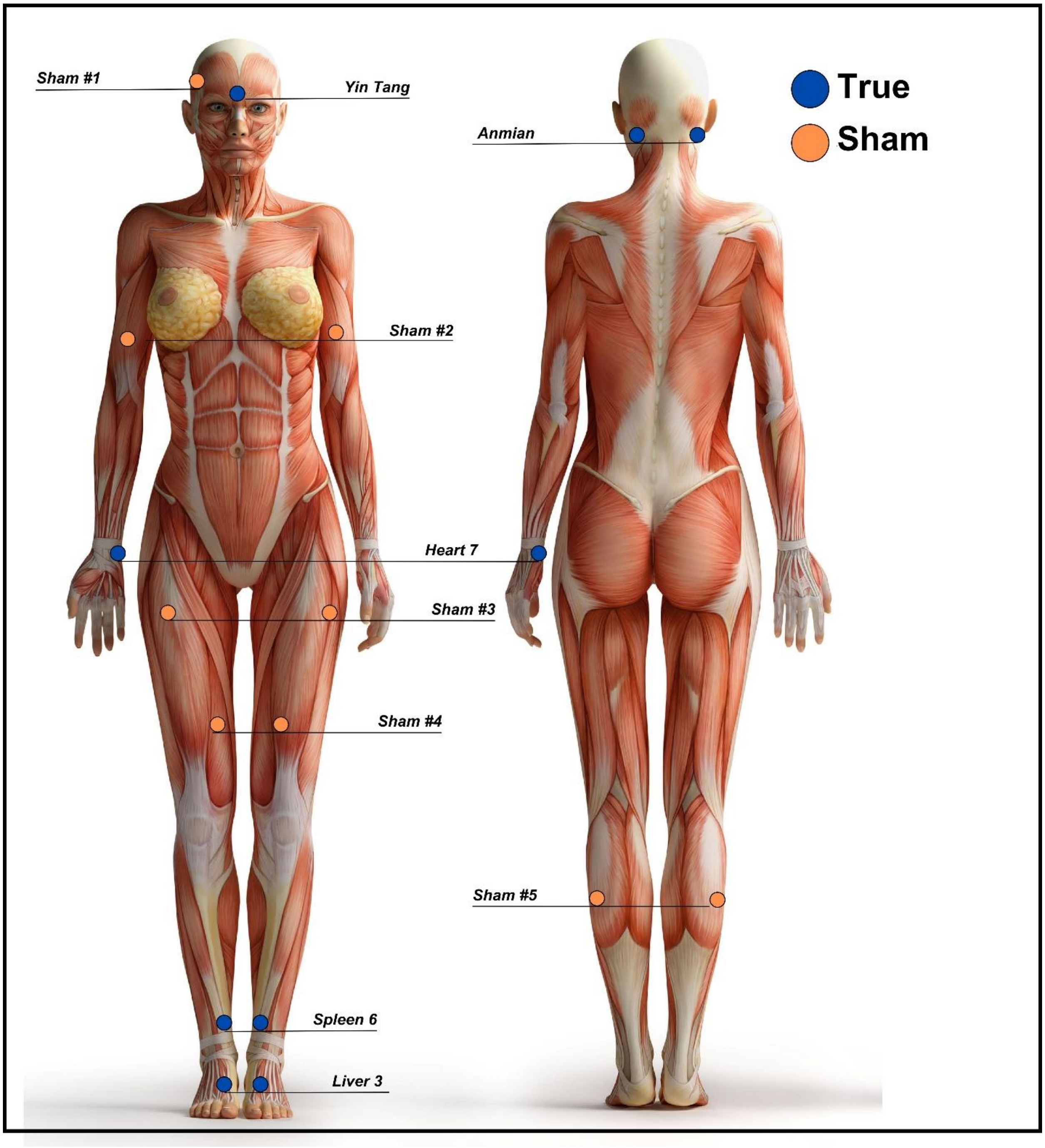

© 2026 Zick SM et al. *JAMA Network Open*.
