# Supplementary material for: Self-Acupressure for Fatigue in Patients Surviving Ovarian Cancer: A Randomized Clinical Trial
Source: JAMA Netw Open. 2026 Feb 5;9(2):e2556357. doi: 10.1001/jamanetworkopen.2025.56357 (PMC12878437; doi:10.1001/jamanetworkopen.2025.56357)
Supplement: Supplement 3. — Data Sharing Statement [file jamanetwopen-e2556357-s003.pdf]

Data Sharing Statement

Zick. Self-Acupressure for Fatigue in Patients Surviving Ovarian Cancer. *JAMA Netw Open*. Published February 02, 2026. doi:10.1001/jamanetworkopen.2025.56357

Data
Additional Information: ClinicalTrials.gov Identifier: NCT03763838
Data available: Yes
Data types: Deidentified participant data
How to access data: szick@umich.edu
When available: With publication

Supporting Documents
Document types: None

Additional Information
Who can access the data: researchers whose proposed use of the data has been approved
Types of analyses: for a specified purpose
Mechanisms of data availability: with a signed data access agreement
